# Exploring Renin-angiotensin System Genes as Novel Prognostic Biomarkers for Oral Squamous Cell Carcinoma

**DOI:** 10.7150/ijms.112735

**Published:** 2025-04-28

**Authors:** Zhengzheng Wu, Can Wang, Jiusong Han, Xiaobing Chen, Jie Wu, Bin Cheng, Juan Wang

**Affiliations:** 1 Hospital of Stomatology, Guangdong Provincial Key Laboratory of Stomatology, Sun Yat-sen University, Guangzhou 510055, Guangdong, People's Republic of China.; 2 Stomatological Hospital, School of Stomatology, Southern Medical University, S366 Jiangnan Boulevard, Guangzhou 510280, Guangdong, People's Republic of China.

**Keywords:** oral squamous cell carcinoma, renin-angiotensin system-related genes, biomarkers, prognosis, risk score

## Abstract

**Purpose:** Recent evidence suggests that the renin-angiotensin system (RAS) is involved in OSCC development. This study aimed to identify RAS-related gene (RASRG) biomarkers associated with OSCC prognosis through integrated bioinformatics analysis.

**Methods:** First, we identified module genes by intersecting differentially expressed genes (DEGs) from the TCGA-OSCC dataset with RASRGs using weighted gene co-expression network analysis (WGCNA). Next, Cox and least absolute shrinkage and selection operator (LASSO) regression analyses were utilized to construct an OSCC risk model. We also created a nomogram incorporating risk scores and relevant clinical variables. Subsequently, receiver operating characteristic (ROC) analysis, Kaplan-Meier (KM) curve analysis, Cox regression analysis, and in vitro experiments were performed to assess the accuracy of the prognostic risk model and nomogram. Furthermore, protein-protein interaction (PPI) network, immune infiltration analysis and functional enrichment analyses were employed to reveal OSCC-related pathogenic genes and underlying mechanisms.

**Results:** A novel OSCC risk model was established consisting of six key genes: *CMA1*, *CTSG*, *OLR1*, *SPP1*, *AQP1*, and *PTX3*. This six-gene signature effectively predicted the prognosis of patients with OSCC and served as a reliable independent prognostic parameter. Protein-protein interaction network analysis identified 5 hub genes and 13 miRNAs. Immune infiltration analysis indicated a possible association of the prognostic features of RASRGs with immunomodulation.

**Conclusion:** In this study, we successfully constructed a risk model based on the six identified RAS-related DEGs as potential predictive biomarkers for OSCC.

## 1. Introduction

Head and neck squamous cell carcinoma (HNSCC) is one of the most common malignant epithelial tumors, with oral squamous cell carcinoma (OSCC) being the most prevalent subtype 1,2. Currently, the standard treatment for OSCC includes surgery, with or without radiotherapy and chemotherapy. Despite advances in early detection and therapeutic strategies, the 5-year survival rate for patients with OSCC remains approximately 50%, largely due to regional relapse and distant metastasis 3. Traditional prognostic indicators, such as tumor staging and grading, fail to distinguish between carcinomas with different biological characteristics within the same histological subgroup. Therefore, there is an urgent need to identify effective prognostic biomarkers and therapeutic targets for OSCC.The renin-angiotensin system (RAS) consists mainly of renin, angiotensinogen (AGT), angiotensin I (Ang I), angiotensin-converting enzyme (ACE), angiotensin II (Ang II), angiotensin II type 1 receptor (AT1R), and angiotensin II type 2 receptor (AT2R) 4. It is an endocrine system that regulates blood pressure homeostasis and electrolyte balance, functioning at both systemic and local levels 5. Accumulating evidence suggests that alterations in the function and activity of the RAS play significant roles in carcinogenesis 6. The ACE/Ang II/AT1R axis promotes angiogenesis, fibrosis, tumor invasion, and metastasis, exerting a tumorigenic role, whereas the ACE-2/Ang 1-7/MASR axis plays an anti-tumorigenic role in various cancers 7. Recent studies have shown dysregulation of RAS components in OSCC, with RAS component expression demonstrated in cancer stem cells of several OSCC subtypes 8,9. Furthermore, genetic variants in renin-angiotensin system-related genes (RASRGS) have been correlated with an increased risk of cancer development and poor prognosis 10. These findings indicate that the RAS is associated with OSCC progression. However, few studies have investigated the prognostic value of RASRGs in patients with OSCC. Moreover, the biological functions of RASRGs in OSCC remain underexplored. In this study, we aimed to identify prognostic biomarkers of RASRGs and develop a prognostic signature for patients with OSCC. We comprehensively analyzed the expression of RASRGs in OSCC and their correlation with OSCC prognosis using The Cancer Genome Atlas (TCGA) and Gene Expression Omnibus (GEO) databases. We constructed a prognostic risk score model consisting of six differentially expressed RAS-related genes (RASRDEGs), namely *CMA1*, *CTSG*, *OLR1*, *SPP1*, *AQP1*, and *PTX3*, to evaluate the predictive value of RASRGs in OSCC. Finally, we experimentally validated the relationship between RASRDEGs and OSCC.

## 2 Materials and Methods

### 2.1 Data source and preprocessing

We downloaded the HNSCC dataset (TCGA-HNSC) from the TCGA database (https://portal.gdc.cancer.gov/) 11. The OSCC dataset (TCGA-OSCC) extracted from this dataset was standardized to fragments per kilobase per million (FPKM) format and used as the test set for analyses. Corresponding clinical data obtained from the UCSC Xena database (https://xena.ucsc.edu/) 12 were presented in Table 1. After excluding samples lacking prognostic information, we obtained the count format sequencing data from 329 OSCC samples with prognostic information and 32 control samples. The GSE30784, GSE23558, and GSE25099 OSCC datasets were downloaded from the GEO database (https://www.ncbi.nlm.nih.gov/geo/) as validation sets 13-19, their specific chip information is shown in Table S1. The combined GEO datasets comprised 251 OSCC samples and 72 control samples after batching (Fig. S1) 20-22. Genes in the TCGA-OSCC dataset and the integrated GEO datasets (Combined Datasets) were intersected, and only the intersecting genes were retained. A total 0f 155 RASRGs were collected from the GeneCards database (https://www.genecards.org/) and related literature 23, as shown in Table S2.

### 2.2 Identification of differently expressed renin-angiotensin related genes

Differentially expressed genes (DEGs) between OSCC and control samples were identified using the R package DESeq2 24 with inclusion criteria |log FC| > 1 and *p* < 0.05 in the TCGA-OSCC dataset. Then RASRDEGs were identified by determining the intersection of the DEGs with RASRGs. The locations of the RASRDEGs on the human chromosomes were analyzed using the R package RCircos. We further analyzed the somatic mutations (SM) and copy number variations (CNV) in OSCC samples from the TCGA-OSCC dataset. The SM profiles were visualized using the R package maftools 25 and GISTIC2.0 26 analysis was performed on the downloaded CNV segments.

### 2.3 Calculation of renin-angiotensin system score and weighted gene association network analysis (WGCNA)

The renin-angiotensin system score (RASScore) for all samples in the TCGA-OSCC dataset was calculated based on the expression matrix of RASRDEGs using the R package GSVA and the ssGSEA algorithm 27. Then receiver operating characteristic (ROC) curve analysis was conducted and the area under the curve (AUC) was calculated using the pROC package. To identify module genes associated with RAS, WGCNA was performed through R package WGCNA 28 and genes with the highest 75% variance were screened. The correlation between RASScore and different modules was measured with a minimum genes number of 100, an optimal soft threshold of 7, a scale-free fit index of 0.94, a module integrated shear height of 0.4 and a minimum distance of 0.2. All genes in modules with |r| > 0.30 were intersected with RASRDEGs and the intersecting genes were identified as module genes.

### 2.4 Expression analysis of module genes in OSCC

Based on their expression levels in OSCC and control samples from the TCGA-OSCC and combined GEO datasets, group comparison plots and ROC curves of module genes 29 were generated. In addition, the consensus clustering method 30 R package ConsensusClusterPlus 31 was used to identify OSCC subtypes. Expression value heatmaps, grouping comparison maps as well as the correlation heatmap and scatter plots were also drawn to further analyze the distribution characteristics of the module genes in different OSCC subtypes.

### 2.5 Construction of prognostic risk model for OSCC

Initially, univariate Cox regression analysis was conducted on module genes using the R package survival 32, and variables with *p* < 0.10 were selected for subsequent least absolute shrinkage and selection operator (LASSO) regression analysis. LASSO regression analysis (family = "cox") was then performed on the previous identified genes using the R package glmnet 33 with an iteration number of 10. Next, multivariate Cox regression analyses were utilized to identify key genes in the risk model. Finally, the risk score was calculated using the following formula:



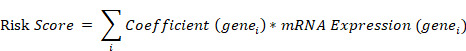



The risk score for OSCC samples from the combined GEO datasets was calculated based on the formula above, and patients were divided into high- and low-risk groups using the median risk score as the dividing point. To further validate the ability of key genes to risk group OSCC samples and their differential expression among different risk groups, we generated group comparison plots and ROC curves 29 based on the expression levels of these genes in OSCC samples from the TCGA-OSCC and combined GEO datasets.

### 2.6 Prognostic analyses of OSCC risk model

To investigate the relationship between gene expression levels and overall survival, OSCC patients were divided into high- and low-risk groups using the median risk score as the cut-off. Based on risk score and overall survival of OSCC patients, time-dependent ROC curves were plotted and the AUC was calculated to predict 1-, 3-, and 5-year survival in OSCC samples from the TCGA dataset. Kaplan-Meier (KM) analysis 34 and log-rank tests were performed to analyze the differences in survival between the two groups. A nomogram was plotted by R package rms 35 based on the results of multivariate Cox regression analysis to demonstrate the interrelationships between the risk score and the clinical information. Additionally, we plotted 1-, 3-, and 5-year calibration curves to evaluate the prognostic accuracy and discriminatory power of the nomogram.

### 2.7 Protein-protein interaction and regulatory network analysis

Patients with OSCC in the TCGA cohort were divided into high- and low-risk groups based on the median risk score. DEGs between the two groups were identified using the R package DESeq2 with inclusion criteria |log FC| > 2.5 and *p* < 0.05. The STRING database (https://string-db.org/) 36 was applied to construct a PPI Network related to RASRDEGs based on DEGs with a minimum required interaction score greater than 0.400. Utilizing Molecular Complex Detection (MCODE) function in Cytoscape software, genes interacted with others in the PPI network were selected as hub genes. The miRNAs associated with hub genes were obtained from the TarBase database (http://www.microrna.gr/tarbase) and the mRNA-miRNA regulatory network was visualized by Cytoscape software. Functional similarity (Friends) analysis was performed using the R package GOSemSim 37 to assess the functional correlation between hub genes. Moreover, we obtained TMB and MSI data from the cBioPortal database (https://www.cbioportal.org/) 38. Mann-Whitney U test (Wilcoxon Rank Sum Test) was performed to evaluate differences in TMB and MSI scores between high- and low-risk groups of OSCC samples from the TCGA-OSCC dataset.

### 2.8 Immune infiltration analyses of prognostic risk model

Enrichment scores representing the relative infiltration abundance of each immune cell was calculated respectively using ssGSE algorithm, and then group comparison plots were drawn to compare the infiltration abundance of 28 immune cells between the high-risk group and the low-risk group. Immune cells with statistically significant difference were selected for subsequent analyses. The correlation between immune cells infiltration abundance was calculated by Spearman algorithm and the results was shown in the correlation heatmap. The correlation between hub genes and immune cells infiltration abundance was also calculated by Spearman algorithm and the results was shown in the correlation bubble plot.

### 2.9 Function enrichment analysis

Gene ontology (GO) 39 and Kyoto Encyclopedia of Genes and Genomes (KEGG) enrichment analyses 40 were conducted to explore the biological functions and pathways associated with prognosis-related DEGs using the R package "clusterProfiler" 41, with an item selection criterion of *p* < 0.05. Additionally, gene set enrichment analysis (GSEA) 42 was performed on all genes in OSCC samples to identify significantly enriched pathways using the R package clusterProfiler 41.

### 2.10 Cell culture

OSCC cell lines (HSC4, HSC6, HN6, and SCC15) and normal oral keratinocytes (NOK) were maintained in our laboratory in Guangzhou, China. HSC4, HSC6, and NOK cell lines were provided by J. Silvio Gutkind (NIH, Bethesda, MD, USA). SCC15 was purchased from the ATCC (Rockville, MD, USA), kindly provided by Professor Xiaoan Tao (Hospital of Stomatology, Sun Yat-sen University, China). HN6 cells were obtained from Cell Bank at the Chinese Academy of Sciences (Shanghai, China). All cells were incubated at 37 °C with 5% CO2. NOK was cultured in keratinocyte serum-free medium (Gibco, USA), supplemented with 25 μg/ml bovine pituitary extract (Gibco, USA), 1 ng/ml epidermal growth factor (Gibco, USA), and 1% penicillin/streptomycin (Gibco, USA). HSC4, HSC6, and SCC15 cells were cultured in Dulbecco's Modified Eagle Medium (DMEM; Gibco) supplemented with 10% fetal bovine serum (FBS; Gibco, USA). HN6 cells were cultured in DMEM/F-12 (Gibco, USA) supplemented with 10% FBS.

### 2.11 Western blot

The cells were washed with ice-cold PBS and lysed using RIPA lysis buffer (Sigma-Aldrich, USA) supplemented with 1% protease and 1% phosphatase inhibitors (Beyotime, China). Next, 5× loading buffer (Beyotime, China) was added to the protein samples, followed by denaturation at 99 °C. The lysates were separated on a 4-20% SDS-PAGE gel and transferred to a 0.22 μm PVDF membrane (Millipore, USA). After blocking with 5% milk, the membranes were incubated overnight at 4 °C with primary antibodies, followed by incubation with species-matched secondary antibodies. The antigen-antibody reaction was visualized using enhanced chemiluminescence (Thermo Fisher, USA). The following antibodies were used: GAPDH (60004-1, 1:3000, Proteintech), AQP1 (ab168387, 1:1 000, Abcam), OLR1 (11837-1, 1:500; Proteintech).

### 2.12 Quantitative reverse transcription-polymerase chain reaction (qRT-PCR)

Total RNA was isolated from the cells using an RNA-Quick Purification Kit (GOONIE, Guangzhou, China). RNA concentration was measured using NanoDrop One (Thermo Fisher, USA). HiScript Ⅲ RT SuperMix for qPCR kit (Vazyme Biotech, Nanjing, China) was used to reverse-transcribe 1 μg of RNA to acquire cDNA. The expression levels of the six key genes were measured using qRT-PCR. SYBR Green-based qPCR analyses were conducted using a QuantStudio 7 Flex System (Thermo Fisher, USA). GAPDH was used as the endogenous control. Relative expression levels were calculated using the comparative threshold cycle equation (2-ΔΔCT). The primer sequences used were as follows: *AQP1*, Forward Primer: 5'- ACCGAGCAGGGTTAATCCCA- 3'; Reverse Primer: 5'-TGTACATCATCGCCCAGTGC-3'; *OLR1*, Forward Primer: 5'-CTTT-GGATGCCAAGTTGCTGAA-3'; Reverse Primer: 5'-GCATCAAAGGAGAAC-CGTCC-3'; *SPP1*, Forward Primer: 5'-AATACCCAGATGCTGTGGCC-3'; Reverse Primer: 5'-ACGGCTGTCCCAATCAGAAG-3'; *GAPDH*, Forward Primer: 5'-CTCCTCCTGTTCGACAGTCAGC-3'; Reverse Primer: 5'-CCCAATACGAC-CAAATCCGTT-3'.

### 2.13 Immunohistochemical (IHC) staining

Twenty patients with OSCC admitted between 2022 and 2024 without prior pre-operative therapy were enrolled and detailed patient information was showed in Table S3. Paraffin-embedded OSCC and para-cancer tissue sections were subjected to immunohistochemical assays with informed consent, following the institutional review board's guidelines. Tissue sections underwent 2h heat treatment at 65 °C, dewaxing with xylene, and alcohol rehydration. Endogenous peroxidase activity was blocked using 3% H_2_O_2_. Citrate-mediated high-temperature antigen retrieval was performed, followed by blocking with goat serum and overnight incubation with primary antibodies. After washing with TBST, secondary antibodies were applied at room temperature. An Apreio AT2 digital whole-slide scanner (Leica, Wetzlar, Germany) was used for slide scanning. The antibodies used included AQP1 (ab168387, 1:1 000, Abcam), OLR1 (11837-1, 1:500; Proteintech), SPP1 (25715-1, 1:500; Proteintech). ImageJ software (National Institutes of Health, Bethesda, Maryland, United States) was used to quantify the stained area.

### 2.14 Statistical analysis

All data processing and analyses were performed using R software (Version 4.3.0). For comparisons between two groups of continuous variables, the significance of differences between normally distributed variables was analyzed using the independent Student's t-test, while non-normally distributed variables were analyzed using the Mann-Whitney U test (Wilcoxon Rank Sum Test). The Kruskal-Wallis test was used to compare three or more groups. Spearman's correlation analysis was used to calculate correlation coefficients between different molecules. All statistical p-values were bilateral, and *p* < 0.05 was considered statistically significant unless otherwise stated.

## 3. Results

### 3.1 Identification of RASRDEGs in OSCC

**Figure 1:**
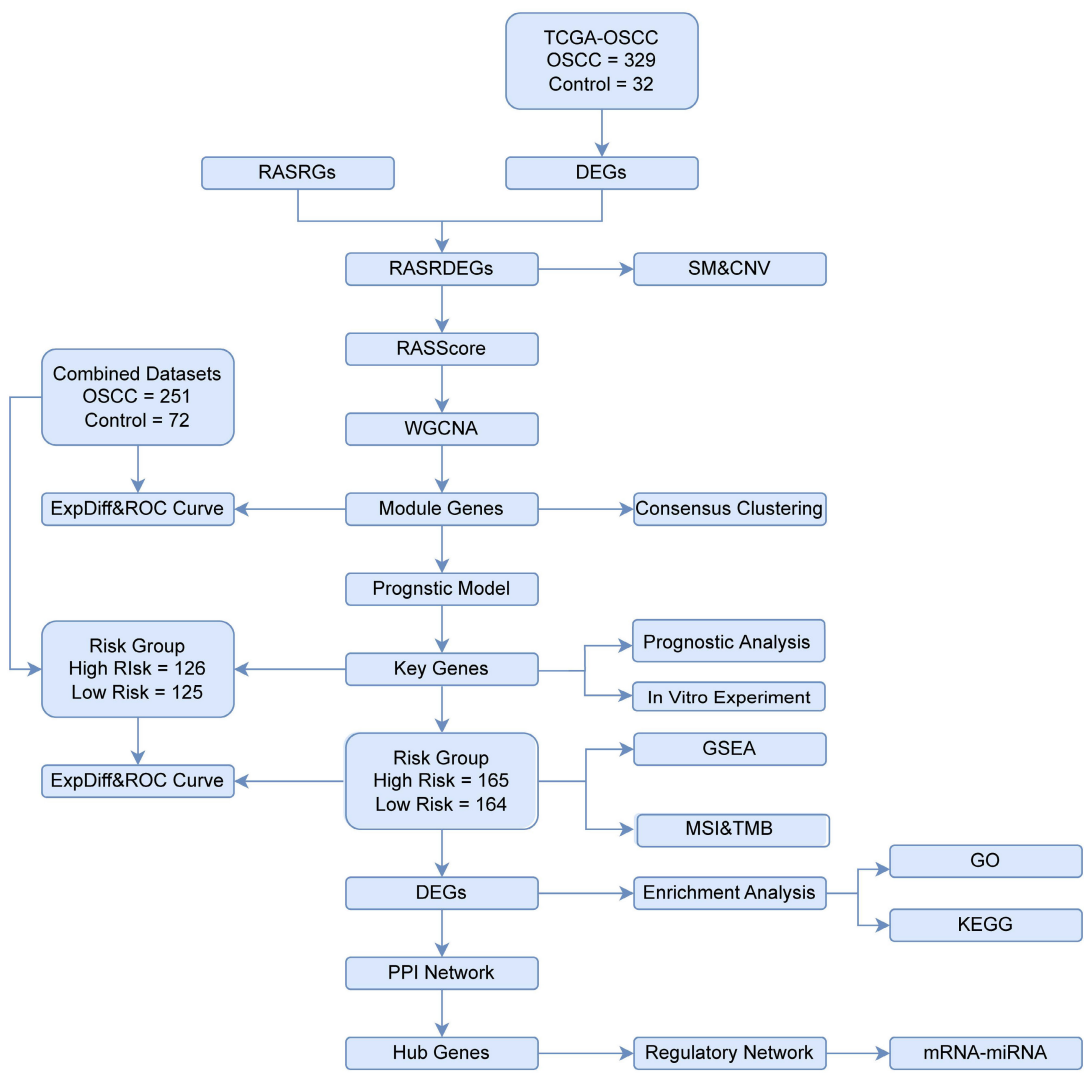
Flowchart illustrating the comprehensive analysis of publicly available data from TCGA and GEO databases.

**Figure 2:**
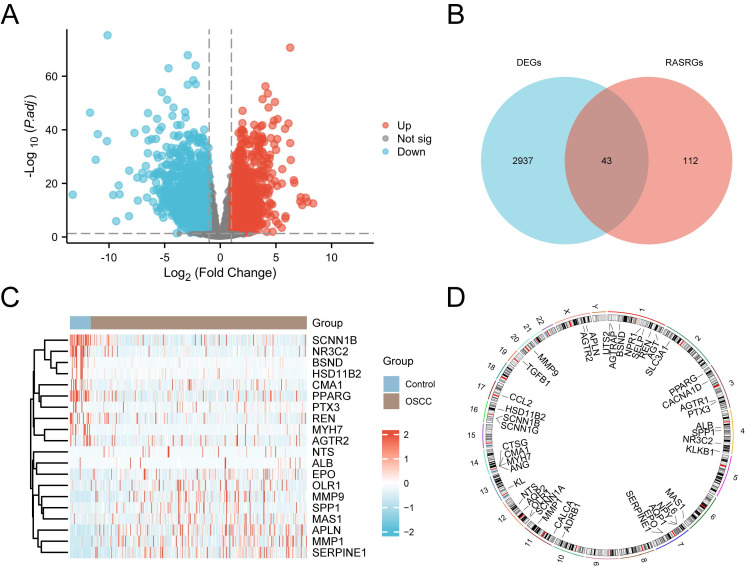
Identification of RASRDEGs in OSCC. **A** Volcano plot depicting differentially expressed genes. **B** Venn diagram showing the intersection of DEGs and RASRGs.** C** Expression heatmap of the top 10 positively and negatively regulated RASRDEGs based on |log FC|. **D** Chromosomal mapping of RASRDEGs.

The study design and data processing were displayed in the flowchart (Fig. 1). In the TCGA-OSCC dataset, we conducted an integrated bioinformatics analysis to explore DEGs, identifying 2,980 DEGs, including 1,262 upregulated and 1,718 downregulated genes (Fig. 2A). A total of 43 RASRDEGs were identified by intersecting the DEGs and RASRGs (Fig. 2B, Table S4). The top 10 positively and negatively regulated RASRDEGs based on |log FC| in the TCGA-OSCC dataset are shown in a heatmap (Fig. 2C). We found most RASRDEGs were located on chromosome 1, including *UTS2*, *AGTRAP*,* BSND*, *NPR1*, *SELP*, *REN*, and *AGT* (Fig. 2D). Additionally, we conducted a mutation analysis of 155 RASRGs in OSCC samples from the TCGA-OSCC dataset. The results revealed six major categories of SM in the RASRGs, with missense mutations being the most common. Single nucleotide polymorphisms (SNPs) were the predominant variant type, and the C-to-T mutation was the most frequent class of single nucleotide variants (SNVs) (Fig. S1A). SM and CNV analysis of the top 10 positively and negatively regulated RASRDEGs in the OSCC samples and found that MYH7 exhibited the highest SM rate of 3% (Fig. S1B-D).

### 3.2 Determining module genes by gene co-expression analysis

**Figure 3:**
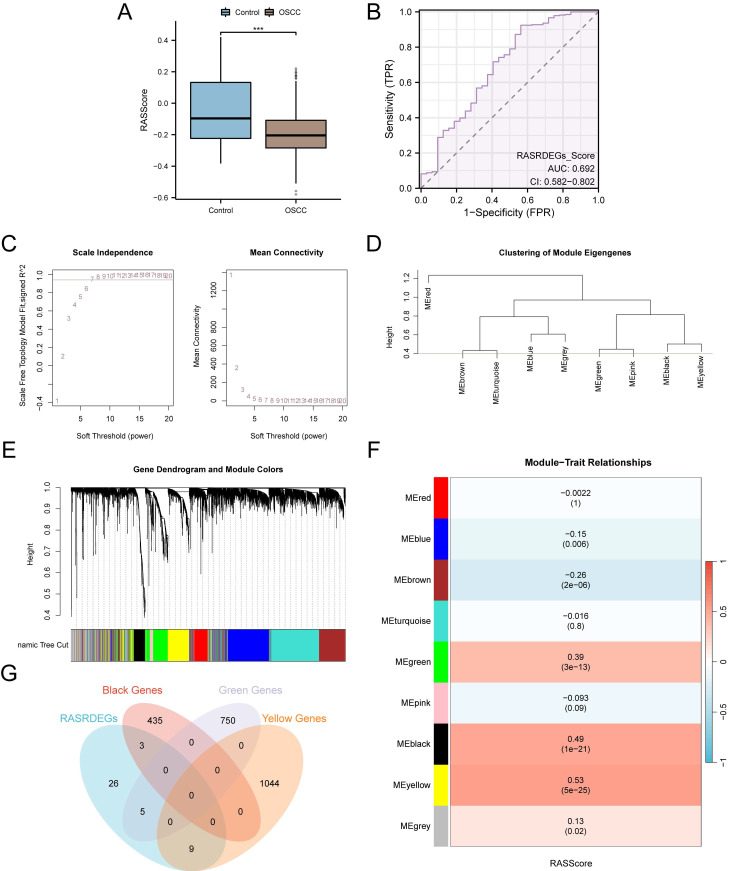
WGCNA of OSCC samples in the TCGA-OSCC dataset. **A** Group comparison diagram of RASScore between OSCC and control samples in the TCGA-OSCC dataset. **B** ROC curve for RASScore in the TCGA-OSCC dataset. **C** Scale-free net-work display of the best soft threshold from WGCNA. (The left panel shows the best soft threshold, and the right panel shows the network connectivity under different soft threshold conditions.)** D-E** Module clustering results of genes with the top 75% variance. (The upper part shows a hierarchical clustering dendrogram, and the lower part shows the gene modules.) **F** Results of correlation analysis between clustered modules and RASScore. **G** Venn diagram of the 43 RASRDEGs and modules MEgreen, MEblack, and MEyellow. ***p < 0.001.

The RASScore for all samples in the TCGA-OSCC dataset was calculated. As shown in the group comparison diagram, RASScore was lower in the OSCC samples than in the Control samples (*p* < 0.001) (Fig. 3A). The results of ROC curves demonstrated that the RASScore had low accuracy in discriminating between patients with OSCC and healthy volunteers (AUC = 0.692) (Fig. 3B).To identify the co-expressed gene modules in OSCC samples from the TCGA-OSCC dataset, WGCNA was performed on the top 75% of DEGs (Fig. 3C). Genes were clustered and labeled with grouping information using hierarchical clustering trees to visualize the relationships between genes and merged modules (Fig. 3D). Using a screening criterion of 0.4, genes with the top 75% variance were clustered into nine modules: MEred, MEblue, MEbrown, MEturquoise, MEgreen, MEpink, MEblack, MEyellow, and MEgrey (Fig. 3E). Correlation analysis between the gene modules and RASScore found three modules with |r| > 0.30, which were selected for further analysis: MEgreen (|r| = 0.39), MEblack (|r| = 0.49), and MEyellow (|r| = 0.53) (Fig. 3F). Finally, 17 module genes were identified by intersecting the 43 RASRDEGs with genes in these three modules: *AGT*, *MYH7*, *NR3C2*, *CCL2*, *CMA1*, *CTSG*, *OLR1*, *SPP1*, *APLN*, *AQP1*, *MMP1*,* MMP9*, *NPR1*, *PTX3*, *SELP*, *SERPINE1*, and *TGFB1* (Fig. 3G).

### 3.3 Expression characteristics of module genes in OSCC

Group comparison plots showed that almost all module genes expressed differently between OSCC and control samples (*p* < 0.01), except for AGT, which showed no statistically significant difference in expression (*p* ≥ 0.05) between the two groups in the combined GEO dataset (Fig. S2A, S3A). The diagnostic efficacy of the module genes in OSCC was further assessed using both the TCGA-OSCC and combined GEO datasets. In the TCGA-OSCC dataset, four module genes (*MMP1*, *MMP9*, *SERPINE1*, and *TGFB1*) exhibited high diagnostic accuracy (AUC > 0.9), 10 module genes (*AGT*, *MYH7*, *NR3C2*, *CCL2*, *CMA1*, *OLR1*, *SPP1*, *APLN*, *AQP1*, and *SELP*) exhibited moderate accuracy (0.7 < AUC < 0.9), and three module genes (*CTSG*, *NPR1*, and *PTX3*) exhibited low accuracy (0.5 < AUC < 0.7) in diagnosing OSCC (Fig. S2B-J). In the combined GEO dataset, five module genes (*NR3C2*, *SPP1*, *MMP1*, *SERPINE1*, and *TGFB1*) displayed high diagnostic accuracy (AUC > 0.9), eight module genes (*CCL2*, *CTSG*, *OLR1*, *APLN*, *AQP1*, *MMP9*, *PTX3*, and *SELP*) displayed moderate accuracy (0.7 < AUC < 0.9), and four module genes (*AGT*, *MYH7*, *CMA1*, and *NPR1*) displayed low accuracy (0.5 < AUC < 0.7) in diagnosing OSCC (Fig. S3B-J). Based on the expression levels of module genes, we group the 329 patients with OSCC in the TCGA-OSCC dataset into two subgroups using the ConsensusClusterPlus package. Subgroup A (cluster 1) consisted of 149 patients, while subgroup B (cluster 2) included 180 patients (Fig. S4A-C). Significant differences between these two subgroups were observed, as shown in the Figure S4D-F. The correlation heatmap revealed that most of the module genes were significantly positively correlated with each other (r > 0, *p* < 0.05) (Fig. S4G), and the top two positively correlated genes are shown in the correlation scatter plot (Fig. S4H-I). The expression patterns of the 17 module genes varied greatly between the two OSCC subtypes. It suggested that RASRDEGs might play a role in defining different clinical subtypes of OSCC, which could be valuable for clinical diagnosis and treatment.

### 3.4 Modeling a RASRGs-related prognostic risk model for OSCC

**Figure 4:**
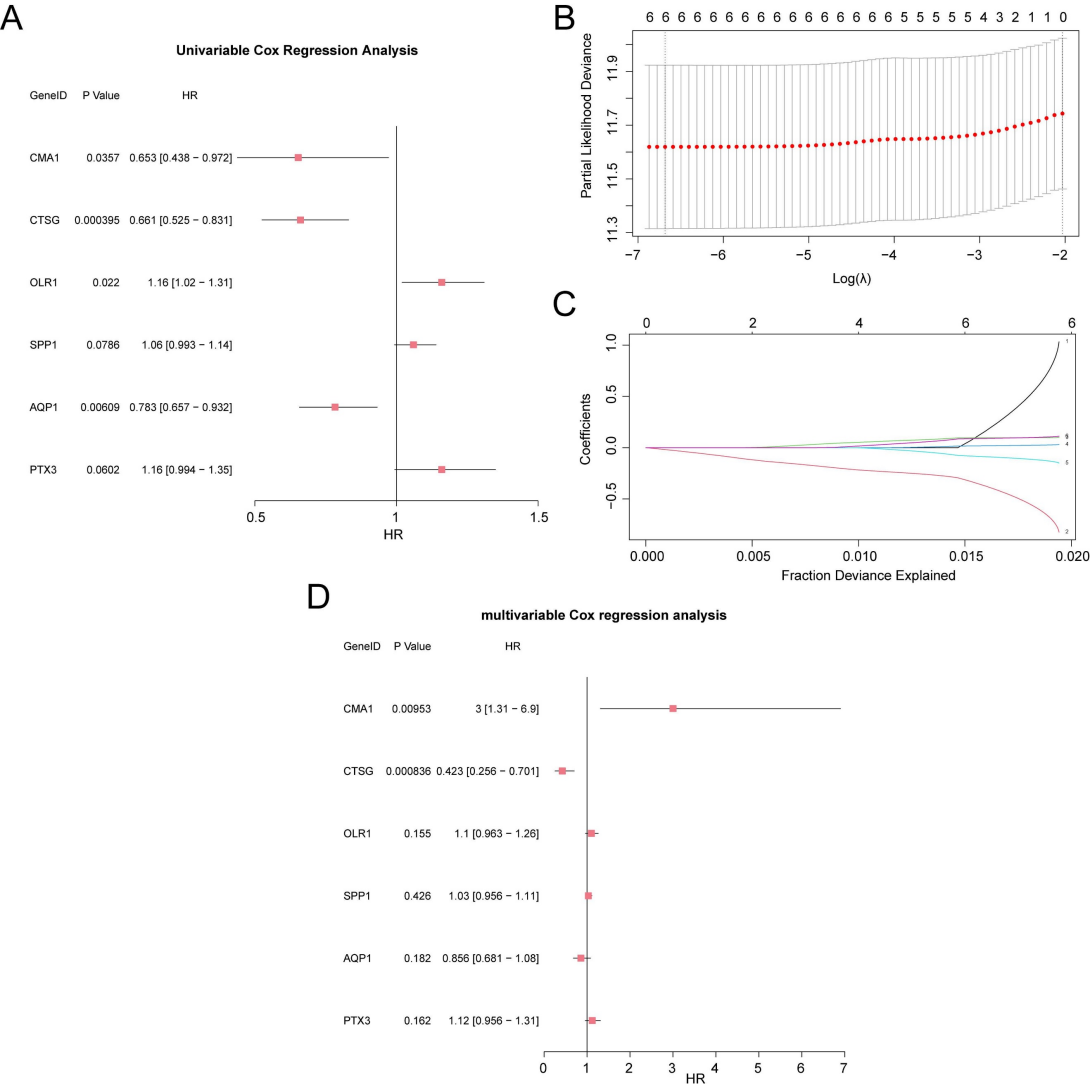
Construction of the RASRGs-related prognostic risk model. **A** Forest plot showing the six key genes in the univariate Cox regression model. **B-C** Plots of the prognostic risk model and variable trajectories from the LASSO regression analysis. **D** Forest plot showing the six key genes in the multivariate Cox regression model.

We combined the module gene expression data with the clinical information of OSCC samples from the TCGA-OSCC dataset and performed a univariate Cox regression analysis. Module genes with *p* < 0.10 were selected for further analysis (Fig. 4A). A LASSO regression analysis was then conducted to build a LASSO regression model (Fig. 4B-D). Based on the optimal number of genes corresponding to the lowest lambda value in the LASSO Cox regression analysis, the model ultimately included six key genes: *CMA1*,* CTSG*, *OLR1*, *SPP1, AQP1*, and *PTX3*. Subsequently, a prognostic risk model was developed, and the risk score was calculated based on the expression levels of these six key genes, weighted by their respective LASSO regression coefficients. The calculation formula is as follows:



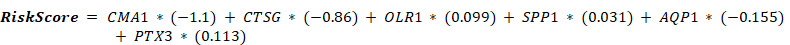



### 3.5 Expression characteristics of key genes in different risk groups

Different expression of the six key genes in both the TCGA-OSCC and combined GEO datasets are presented in group comparison plots (Fig. S5A-B). The expression levels of *AQP1*, *CMA1*, and *CTSG* were lower, whereas those of *OLR1*, *PTX3*, and *SPP1* were higher in the high-risk group compared to the low-risk group (*p* < 0.05). However, no statistically significant difference was observed in the expression of *CMA1* between the high- and low-risk groups in the combined GEO dataset (*p* ≥ 0.05). Furthermore, ROC analysis was performed to evaluate the discriminative ability of the six key genes in the OSCC risk groups. In the TCGA-OSCC dataset, *CTSG* demonstrated high accuracy (AUC > 0.9) in risk grouping of OSCC samples, whereas *CMA1* and *AQP1* demonstrated moderate accuracy (0.7 < AUC < 0.9), and *OLR1*, *SPP1*, and *PTX3* showed low accuracy (0.5 < AUC < 0.7) (Fig. S5C-E). In the combined GEO dataset, *CTSG* and *AQP1* showed moderate accuracy (0.7 < AUC < 0.9) in risk grouping of OSCC samples, whereas *CMA1*, *OLR1*, *SPP1*, and *PTX3* demonstrated low accuracy (0.5 < AUC < 0.7) (Fig. S5F-H).

### 3.6 Prognostic performance analysis of RASRGs-related risk model

**Figure 5:**
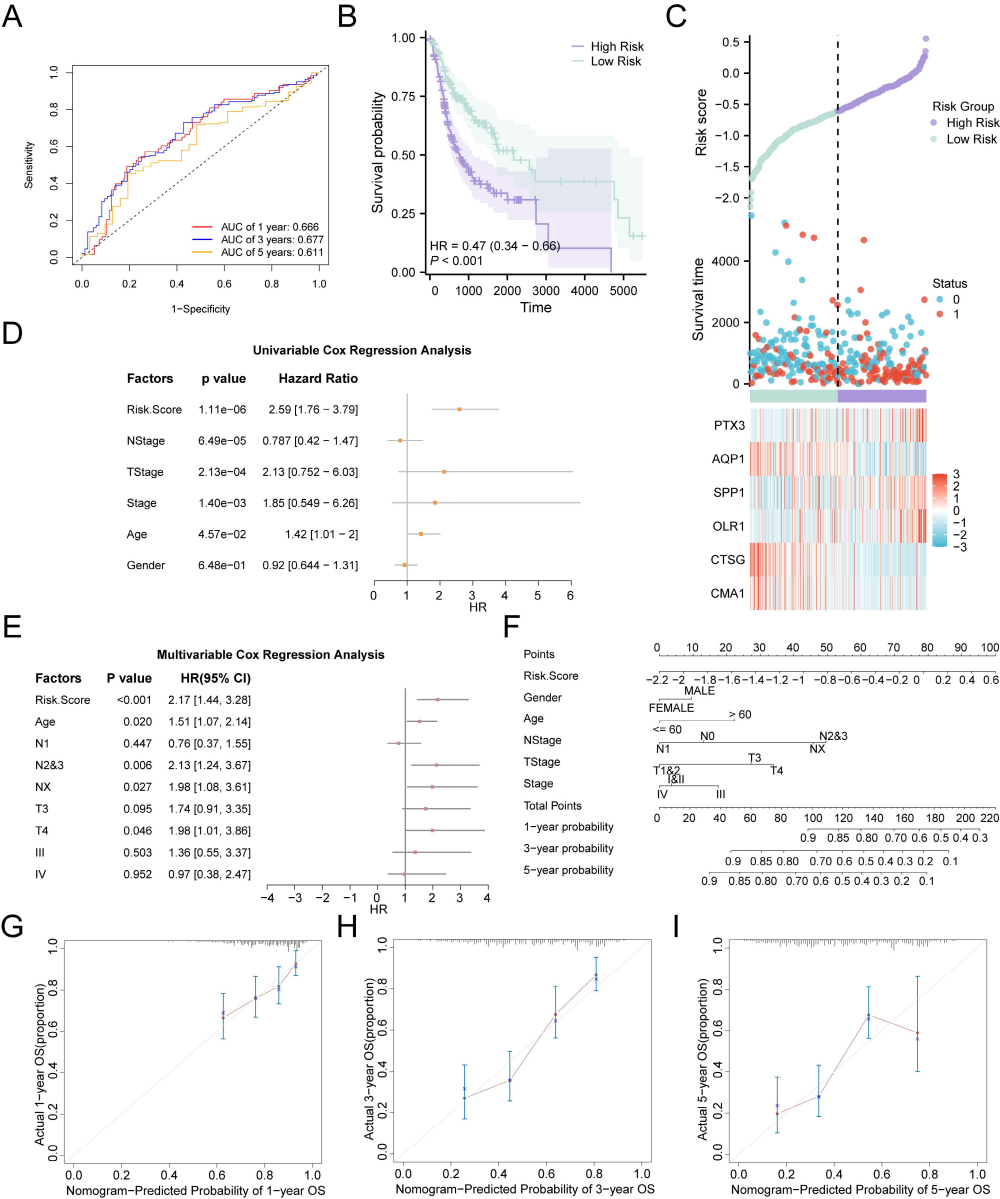
Prognostic analysis of the RASRGs-related risk model. **A** Time-dependent ROC curve for OSCC samples in the TCGA-OSCC dataset. **B** Prognostic Kaplan-Meier (KM) curves for high-risk and low-risk OSCC groups. **C** Risk factor plot of the prognostic risk model for OSCC. **D-E** Forest plots of risk score and clinical information in univariate and multivariate Cox regression models.** F** A nomogram integrating risk scores and clinical parameters for precision prediction. **G-I** Calibration curves of the prognostic risk model for 1-, 3-, and 5-year overall survival (OS).

The AUCs of time-dependent ROC curves for the 1-, 3-, and 5-year survival probabilities were all greater than 0.6 (0.666, 0.677, and 0.611, respectively), indicating that the risk model demonstrated promising prognostic predictive ability in the validation cohort (Fig. 5A). The risk score for each patient was calculated using the prognostic model, and patients with OSCC were divided into high- and low-risk groups, with the median risk score serving as the cut-off point. Patients in the high-risk group had lower survival rates than those in the low-risk group, as shown by the Kaplan-Meier curves (*p* < 0.001) (Fig. 5B). Distribution of risk scores and survival data in the two risk groups was illustrated using a risk factor plot (Fig. 5C). Notably, univariate Cox regression analysis revealed that the risk score was significantly associated with poorer OS (overall survival) in OSCC (HR = 2.59, 95% CI: 1.76-3.79, *p* < 0.01) (Fig. 5D). Further multivariate Cox regression analysis identified the risk score, age, N stage, and T stage as independent prognostic indicators for patients with OSCC (*p* < 0.05) (Fig. 5E, Table S5). To further investigate the prognostic predictive value of the multi-gene risk model for OSCC, a nomogram was constructed based on the Cox regression analysis (Fig. 5F). The results demonstrated that the utility of the risk score was substantially higher than that of the other variables, while sex had the lowest utility. Additionally, calibration analysis showed a high degree of agreement between the nomogram predictions and actual observations for the 3-year OS predictive probabilities (Fig. 5G-I). In conclusion, the RASRG-related prognostic risk model is both reliable and valid for predicting the prognosis of patients with OSCC.

### 3.7 Verification of expression levels of prognostic signature genes in OSCC tissues and cell lines

**Figure 6:**
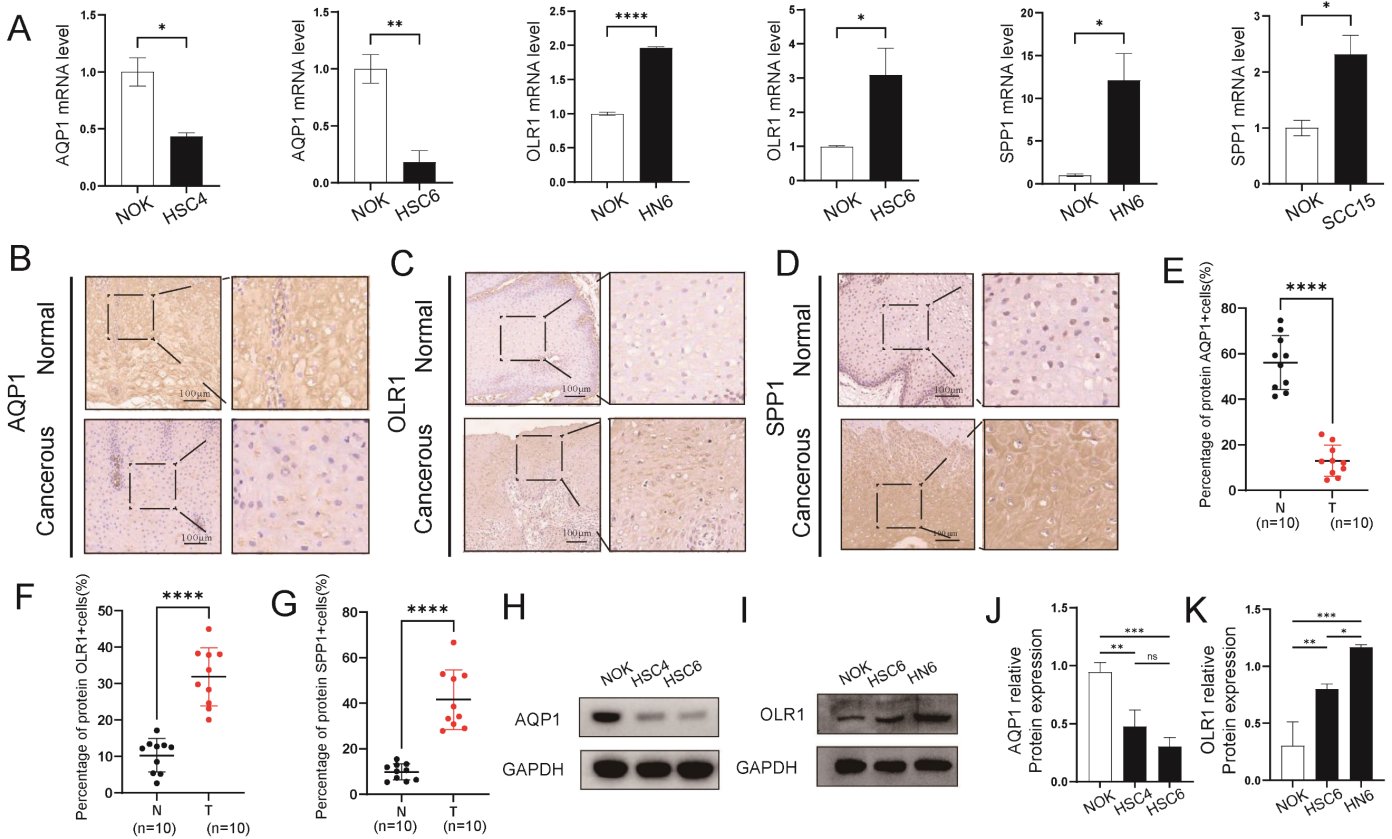
Validation of the expression patterns of key genes in OSCC tissues and cell lines. **A** Comparative qPCR analysis illustrating the expression disparities of key risk genes, including *AQP1*, *OLR1*, and *SPP1*, between normal oral epithelial cells (NOK) and OSCC cell lines. **B-G** Immunohistochemical staining of *AQP1* (**B**, **E**), *OLR1* (**C**, **F**), and *SPP1* (**D**, **G**) in OSCC cancerous tissues and adjacent normal tissues. **H-K** Western blot analysis of *AQP1* (**H**, **J**) and *OLR1* (**I**, **K**) levels in NOK and OSCC cell lines. * *p* < 0.05, ** *p* < 0.01, *** *p* < 0.001, **** p < 0.0001.

To confirm the reliability and accuracy of the results obtained from the bioinformatics analysis, we validated the mRNA and protein levels of key genes in OSCC samples. We selected three key genes (*AQP1*, *OLR1*, and *SPP1*) with higher discriminative ability (AUC > 0.7) for OSCC in both the TCGA-OSCC and combined GEO datasets as validation genes. Our results revealed that *AQP1* exhibited low expression in OSCC cell lines, whereas the expression levels of *OLR1* and *SPP1* were higher in OSCC cell lines compared to normal cells (Fig. 6A, H-K). Additionally, the expression levels of *AQP1*, *OLR1*, and *SPP1* in cancerous and para-cancerous tissues were significantly different. The IHC results showed that the expression trends of these genes in tissues were consistent with those observed in the cell lines (Fig. 6B-G). Overall, these results are consistent with the bioinformatics analysis.

### 3.8 Construction of PPI network and regulatory network

**Figure 7:**
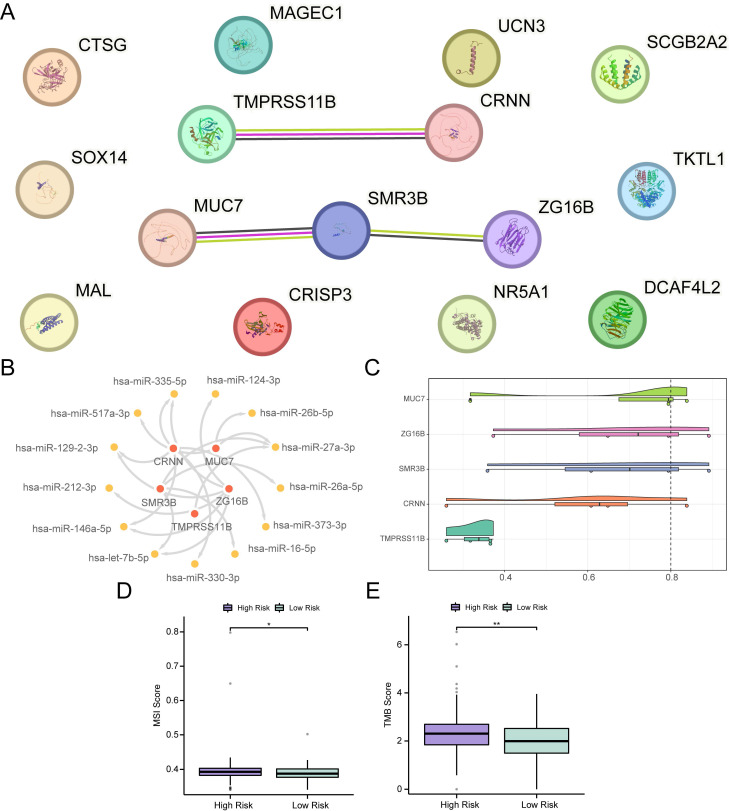
PPI and regulatory network, MSI, and TMB analyses. **A** PPI network of 15 DEGs. **B** mRNA-miRNA regulatory network of hub genes. **C** Cloud and rain diagram of friend analysis. **D-E** Group comparison plots of MSI scores (**D**) and TMB scores (**E**) between different OSCC risk groups. * *p* < 0.05, ** *p* < 0.01.

Differential expression analysis was conducted between the high- and low-risk groups of OSCC samples in the TCGA-OSCC dataset, resulting in the identification of 15 DEGs, including *CTSG*, *NR5A1*, *MUC7*, *CRISP3*, *ZG16B*, *CRNN*, *TKTL1*, *MAL*, *TMPRSS11B*, *SCGB2A2*,* DCAF4L2*, *SOX14*, *MAGEC1*, *UCN3*, and *SMR3B*. Among these, six genes were upregulated and nine were downregulated (Fig. S7A-B). A PPI network was constructed for these 15 DEGs, and five hub genes were identified in the PPI network, including *TMPRSS11B*, *CRNN*, *MUC7*, *SMR3B*, and *ZG16B* (Fig. 7A). Subsequently, 13 miRNAs associated with the five hub genes were obtained from the TarBase database to construct an mRNA-miRNA regulatory network (Fig. 7B, Table S6). Additionally, friend analysis of the hub genes revealed that *MUC7* had the strongest correlation with the other hub genes (Fig. 7C). *MUC7* was the gene closest to the cut-off value (cut-off value = 0.80), suggesting that it may play a central role in the biological processes of OSCC.Furthermore, mutation analysis showed that the low-risk group exhibited lower MSI and TMB scores than the high-risk group (Fig. 7D-E) (*p* < 0.05), indicating a potential link between the prognostic features of RASRGs and tumor somatic mutation trends.

### 3.9 Immune infiltration analysis and Potential molecular characteristics of prognostic risk model

**Figure 8:**
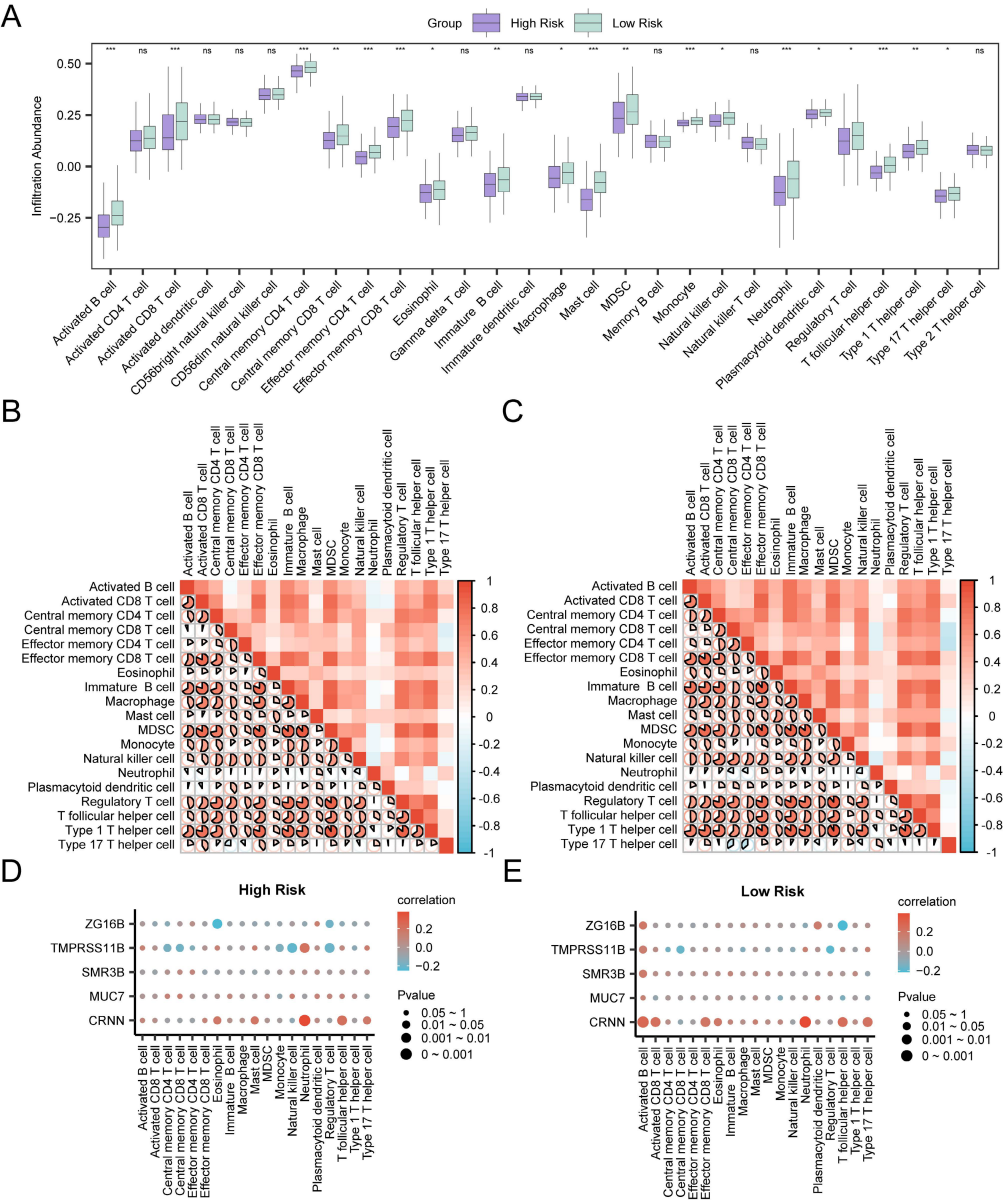
Immune Infiltration analyses of Risk Groups. **A** Group comparison plots of immune cells in the high-risk group and the low-risk group of OSCC samples. **B-C** Correlation heatmaps of immune cells infiltration abundance in the high-risk group (**B**) and the low-risk group (**C**) of OSCC samples. **D-E** Bubble plots of correlation between immune cell infiltration abundance and Hub Genes in the high-risk (**D**) and low-risk (**E**) groups of OSCC.

Infiltration abundance of immune cells in the high-risk group and the low-risk group was calculated through ssGSEA algorithm. As shown in the group comparison group (Fig. 8A), the infiltration abundance of nineteen immune cells in the low-risk group was statistically higher than those in the high-risk group (*p* < 0.05), including activated B cell, activated CD8 T cell, central memory CD4 T cell, effector memory CD4 T cell, effector memory CD8 T cell, mast cell, monocyte, neutrophil, T follicular helper cell, central memory CD8 T cell, immature B cell, MDSC, type 1 T helper cell, eosinophil, macrophage, natural killer cell, plasmacytoid dendritic cell, regulatory T cell and type 17 T helper cell. The degree of immune infiltration of nine other immune cells was not statistically significant between the two groups. In both high-risk group and low-risk group, most of the immune cells showed relatively strong positive correlation (Fig. 8B-C). In addition, the correlations between hub genes and the infiltration abundance of immune cells were also assessed. In the high-risk group, CRNN showed the strongest positive correlation with neutrophil (r = 0.383, *p* < 0.05), and ZG16B showed the strongest negative correlation with eosinophil (r = -0.25, *p* < 0.05) (Fig. 8D). In the low-risk group, CRNN showed the strongest positive correlation with neutrophil (r = 0.387, *p* < 0.05), and ZG16B showed the strongest negative correlation with T follicular helper cell (r = -0.215, *p* < 0.05) (Fig. 8E). This evidence suggested a possible association of the prognostic characteristics of RASRGs and immune regulation, in which hub genes may play a regulatory role.

### 3.10 Potential molecular characteristics of prognostic risk model

A total of 15 DEGs were included in the GO and KEGG enrichment analyses. The results indicated that these DEGs were primarily involved in the biological processes (BP) of hormone-mediated signaling pathway. They were also enriched in molecular functions (MF) of serine-type endopeptidase activity, serine-type peptidase activity, serine hydrolase activity, caspase binding, and peptidase regulator activity. In terms of KEGG pathways, the DEGs were enriched in the RAS, neuroactive ligand-receptor interaction, pentose phosphate pathway, cortisol synthesis and secretion, and biosynthesis of amino acids (Fig. S7C-G). More detailed results of the GO and KEGG enrichment analyses were shown in Table S7. The RAS pathway enrichment analysis was visualized using the R package Pathview (Fig. S8).Additionally, GSEA was performed to investigate potential pathway associated with RAS in OSCC, and the specific results were shown in Table S8 (Fig. S9A). As the results indicated, all genes in OSCC samples were significantly enriched in several key signaling pathways, including the PI3K-Akt signaling pathway (Fig. S9B), JAK-STAT signaling pathway (Fig. S9C), FceRI-mediated NF-κB activation (Fig. S9D), IL-2 pathway (Fig. S9E), and FceRI-mediated MAPK activation (Fig. S9F). These enriched BP, MF and other biological pathways may serve as potential therapeutic targets for OSCC treatment.

## 4. Discussion

OSCC remains an intractable disease with a low 5-year survival rate, primarily because most patients are diagnosed at an advanced stage, which is strongly associated with poor prognosis. Distinguishing patients with different biological characteristics will facilitate early diagnosis and intervention, as well as allow for stratified prognosis assessment, which can significantly improve patient survival. While RAS has recently been linked to OSCC, the functional landscape and mechanistic underpinnings of RASRGs in this malignancy await systematic investigation. In the present study, we applied a bioinformatics approach to elucidate the prognostic value of RASRGs in OSCC and constructed a RASRG-related risk model comprising six key genes (*CMA1*, *CTSG*, *OLR1*, *SPP1*, *AQP1*, and *PTX3*). This model was rigorously validated using data from 329 patients in the TCGA-OSCC dataset.

The key genes *CMA1*, *CTSG*, *OLR1*, *SPP1*, and *PTX3* have been identified as potential prognostic markers for OSCC in previous studies. *CMA1* encodes a serine protease that activates angiotensin II, a key component of the RAS 43. *CMA1* is expressed at low levels in OSCC cells and tissues, which may influence OSCC progression by regulating Ang II synthesis through ACE/Ang II/AT1R axis or ACE/Ang II/AT2R axis 44,45. Its abnormal expression has been reported in prostate cancer 46. *CTSG* inhibits HNSCC proliferation and metastasis in vivo and in vitro, which has the potential to be an oncogenic factor for HNSCC by focusing on the JAK2/STAT3 signaling pathway 47*. CTSG* is considered a potential immune-related biomarker in OSCC, involved in host immune defense, tumor angiogenesis, and metastasis 48-50, and has been targeted for immunotherapy in acute myeloid leukemia (AML) 51. Low expression levels of *CMA1* and *CTSG* are associated with poor OS 44,48-50. In contrast, the expression of *OLR1* and *SPP1* is significantly elevated in OSCC cell lines and tissues, promoting OSCC development and correlating with poor prognosis 52-56. *OLR1* may affect EMT, invasion, stemness, and proliferative activity of HNSCC 52-54*.* What's more, *OLR1* expression positively correlates with immune-suppressive cell infiltration and immune checkpoint molecules, while negatively correlating with effector T cells, suggesting its correlation with immune-suppressive microenvironment 57. *SPP1*, also known as OPN, functions as a crucial adhesion protein and plays a major role in numerous tumors. *SPP1* facilitates proliferation, metastasis, angiogenesis, and disease progression 55,56. A recent study found that *SPP1*+ macrophages increase the secretion of TNF-α and IL-1β via the NF-kappa B pathway to promote HNSCC cell proliferation, and TNF-α and IL-1β in turn upregulate the expression of OPN in tumor cells and macrophages 58.* PTX3* is involved in cancer-associated inflammation, upregulated in the surgical margins of advanced OSCC, and correlates with cancer recurrence and progression 59. In addition, *PTX3* can affect cell proliferation, cycle and apoptosis, and may also affect the expression of HLA system-related proteins in esophageal squamous cell carcinoma (ESCC) 60. Recently, *AQP1* has been reported as a distinctive prognostic factor in various cancers, including breast, cervical, colorectal, hepatocellular, lung, renal, and squamous cell carcinomas 61,62. *AQP1* has traditionally been recognized as a water channel protein, and many studies have shown its association with carcinogenesis, metastasis, poor prognosis, lymph node metastasis, and cellular migration 61-63. However, the role of *AQP1* in HNSCC remains controversial. On one hand, *AQP1* has been suggested to act as a tumor suppressor inhibiting the growth of HNSCC 64,65. On the other hand, *AQP1* is highly expressed in aggressive basaloid-like oro-hypopharynx squamous cell carcinomas with poor prognosis 66. The downregulation of *AQP1* in OSCC may suppress tumor cell motility, yet its specific biological functions in this malignancy require further investigation. In this study, for the first time, we found that *AQP1* is downregulated in OSCC tissues and cell lines, and we demonstrate that *AQP1* may serve as a potential prognostic biomarker for OSCC. The roles of the five key genes in OSCC were consistent with previous reports, with *CMA1* and *CTSG* acting as protective genes and *OLR1*, *PTX3*, and* SPP1* acting as risk-promoting genes. Additionally, *AQP1* appears to function as a protective gene against OSCC. Although several key genes have been previously implicated as prognostic markers or functionally relevance in OSCC or other cancers, our study is the first to link these genes to RAS abnormalities and suggests that they may have important role in shaping the immunosuppressive microenvironment, highlighting their combined potential as therapeutic target.

Our study identified several clinically actionable applications of the RAS-related gene signature. First, the risk model has strong potential for prognostic stratification that could complement existing TNM staging by capturing biological aggressiveness beyond the anatomical range. With more accurate risk stratification, high-risk patients could be treated more aggressively, whereas low-risk patients might be spared overtreatment. Second, clinical applications could also extend to early detection strategies, with noninvasive testing of RAS-related biomarkers in saliva or other body fluid as liquid biopsy to screen high-risk populations. What's more, our findings support exploring repurposed RAS-modulating drugs, such as ACE inhibitors and angiotensin receptor blockers (ARBs). These drugs had shown promise in preclinical cancer models, and their role in OSCC required experimental exploration 67-69. Our immune infiltration analysis showed that high-risk tumors have immunosuppressive features, suggesting a possible synergy between RAS modulation and immunotherapy-a hypothesis worthy of clinical investigation. To facilitate clinical implementation, we outline three key translational steps: prospective validation in multicenter cohorts, functional studies to elucidate mechanisms and testing the potential value of RAS-modulating drugs in OSCC.

Although no direct comparisons of current standard prognostic tools (e.g., TNM staging) were performed in this paper, we analyzed baseline TNM staging in the TCGA dataset to assess its role in prognostic modeling. In our study, a multivariate Cox regression analysis showed that the risk score as well as age, N-staging and T-staging were significant (*p* < 0.05) in prognosis and the utility of the risk score was substantially higher than that of the other variable. It tentatively suggested that our risk score can be combined with traditional TNM staging to provide a more comprehensive assessment of patient prognosis.

Our study indicated that the hub gene* MUC7* might play a critical role in OSCC, as suggested by PPI network analysis. It was found that *MUC7* expression was down-regulated in the high-risk group, suggesting that low expression of *MUC7* may be detrimental to the survival of patients with OSCC. The present finding is concordant with the results of previous study proposing that high expression of *MUC7* is associated with better survival in patients with HNSCC 70. It is noteworthy that most immune cells including neutrophil, eosinophil, activated B cell, activated CD8 T cell and natural killer cell showed lower infiltration abundance in the in the high-risk group, suggesting extensive RAS-related immunosuppressive microenvironment might exist in the high-risk group. Further functional enrichment analysis revealed that a larger number of DEGs were enriched in the BP of the hormone-mediated signaling pathway and the renin-angiotensin system, reiterating the connection between RAS and OSCC prognosis. Additionally, several key pathways were identified in GSEA, including the PI3K-Akt signaling pathway, JAK-STAT signaling pathway, FceRI-mediated NF-κB activation, IL-2 pathway, and FceRI-mediated MAPK activation. Previous studies have shown that the PI3K-Akt-mTOR, JAK-STAT3, and MAPK pathways are downstream of AT1R, which promotes cell proliferation and cancer progression 6,71-73. NF-κB synergizes with Ang II in cancer development by regulating metastasis and angiogenesis 74-76. In our study, all of these signaling pathways were downregulated in the high-risk group compared to the low-risk group. These findings can be interpreted in several ways. First, OSCC samples from the high-risk group may represent more advanced tumor stages, where cells may reduce the activity of pathways that promote cell proliferation and survival in order to adapt to unfavorable microenvironmental conditions. Second, negative NES values may be associated with poor prognosis in OSCC patients. Tumor cells may downregulate these signaling pathways to avoid recognition and attack by the immune system. The IL-2 pathway, which was the first immunotherapy approved for cancer treatment nearly 30 years ago by the U.S. Food and Drug Administration (FDA) 77, plays a crucial role in counteracting the dysregulated immune system by targeting regulatory T cells and enhancing antitumor responses through effector, memory, and natural killer cells 78,79. In our study, IL-2 was downregulated in the high-risk group, indicating that low expression of IL-2 may contribute to a worse prognosis. These enrichment pathways may be significant molecular mechanism for OSCC to form an immunosuppressive microenvironment and escape from immune supervision.

Our study identified RAS-related biomarkers and established a prognostic risk model for OSCC, however, there are still several limitations. First, the retrospective nature of the study and the modest sample size might limit the generalizability of the risk model. Second, clinical heterogeneity (e.g., variations in tumor stage, treatment history, and comorbidities) was not fully adjusted in the multivariate analysis. Larger prospective cohorts are needed to validate the model's robustness. Third, although we validated key RAS genes at the mRNA and protein level, functional experiments (e.g., gene knockdown/overexpression) were not performed to establish their causal roles in OSCC progression. Additionally, the results of miRNA-mRNA and PPI networks still need experimental validation of the expression patterns and binding specificity. Ultimately, the risk model's utility in guiding personalized therapy (e.g., RAS-targeted drugs) also needed lots of prospective trials to assess its predictive value for treatment response.

## 5. Conclusion

In conclusion, to the best of our knowledge, our study is the first to identify the significant impact of renin-angiotensin-related genes on the clinical prognosis of OSCC. We constructed a six-gene (*CMA1*,* CTSG*, *OLR1*, *SPP1*, *PTX3*, and *AQP1*) prognostic risk model and verify its accuracy and universality in predicting the prognosis of OSCC. Our findings may facilitate personalized treatment strategies for patients with OSCC at different risks.

## Supplementary Material

Supplementary figures and tables.

## Figures and Tables

**Table 1:** Characteristics of patients with OSCC in TCGA database

Characteristics	Overall
Age, n (%)	
> 60	154 (52.2%)
<= 60	141 (47.8%)
Gender, n (%)	
MALE	203 (68.8%)
FEMALE	92 (31.2%)
N Stage, n (%)	
N0	115 (39%)
N2&3	107 (36.3%)
NX	24 (8.1%)
N1	49 (16.6%)
T Stage, n (%)	
T4	109 (36.9%)
T1&2	121 (41%)
T3	65 (22%)
Stage, n (%)	
IV	165 (55.9%)
I&II	70 (23.7%)
III	60 (20.3%)

OSCC: oral squamous cell carcinoma

## Abbreviations

ACE: angiotensin converting enzyme
AUC: area under the ROC curve
BP: biological process
CDF: cumulative distribution function
CNV: copy number variation
DEGs: differentially expressed genes
DMEM: Dulbecco's modified eagle medium
DOI: digital object identifier
FBS: fetal bovine serum
FPKM: fragments per kilobase per million
GEO: gene expression omnibus
GO: gene ontology
GSEA: gene set enrichment analysis
HNSCC: head and neck squamous cell carcinoma
KEGG: Kyoto Encyclopedia of Genes and Genomes
LASSO: least absolute shrinkage and selection operator
MCODE: molecular complex detection
MF: molecular functions
MSI: microsatellite instability
NOK: Normal oral keratinocytes
OS: overall survival
OSCC: oral squamous cell carcinoma
PPI: protein interaction
qRT-PCR: quantitative reverse transcription-polymerase chain reaction
RAS: renin-angiotensin system
RASRG: RAS-related gene
RASRDEG: renin-angiotensin system-related differentially expressed gene
ROC: receiver operating characteristic
SM: somatic mutations
SNP: single nucleotide polymorphism
SNV: single nucleotide variant
TCGA: the cancer genome atlas
TMB: tumor mutation burden
WGCNA: weighted gene co-expression network analysis
