# Supplementary material for: Exploring Renin-angiotensin System Genes as Novel Prognostic Biomarkers for Oral Squamous Cell Carcinoma
Source: Int J Med Sci. 2025 Apr 28;22(10):2470–87. doi: 10.7150/ijms.112735 (PMC12080578; doi:10.7150/ijms.112735)
Supplement: Supplementary file 1 — Supplementary figures and tables. [file ijmsv22p2470s1.pdf]

Supplemental Materials

Supplemental Figures

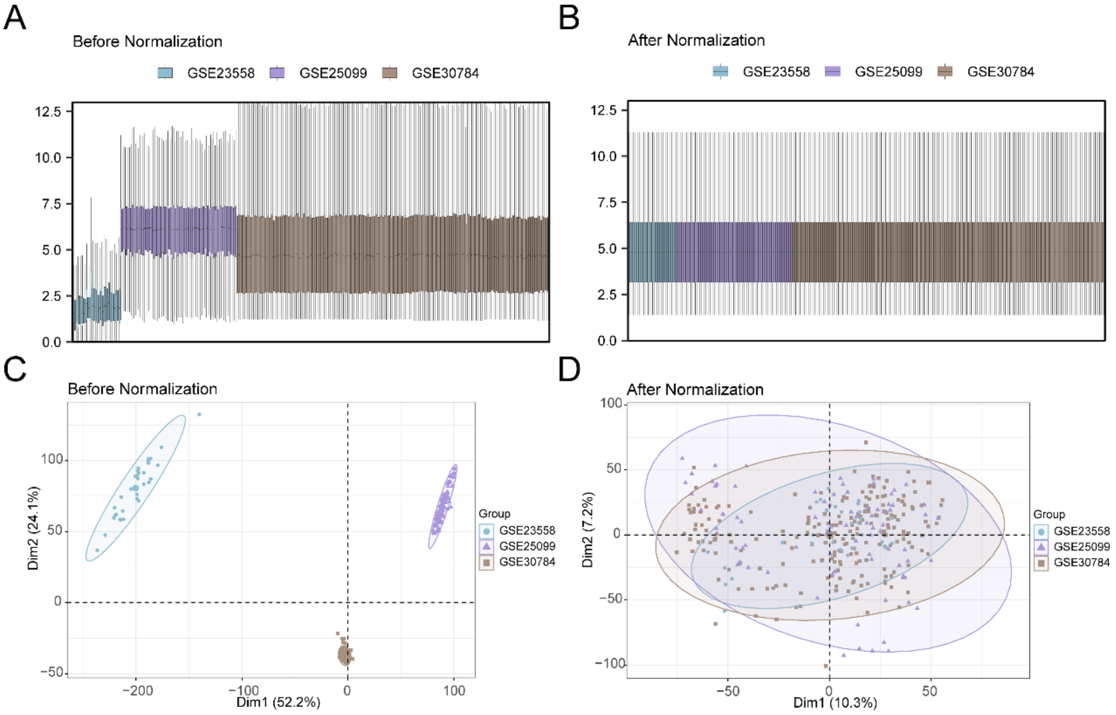

Fig. S1 Batch effects removal of GSE30784, GSE23558 and GSE25099. **A** Box plot of Combined GEO Datasets distribution before batch removal. **B** Distribution boxplots of Combined GEO Datasets after batch removal. **C** PCA plot of the Combined GEO Datasets before batch removal. **D** PCA plot of Combined GEO Datasets after batch removal.

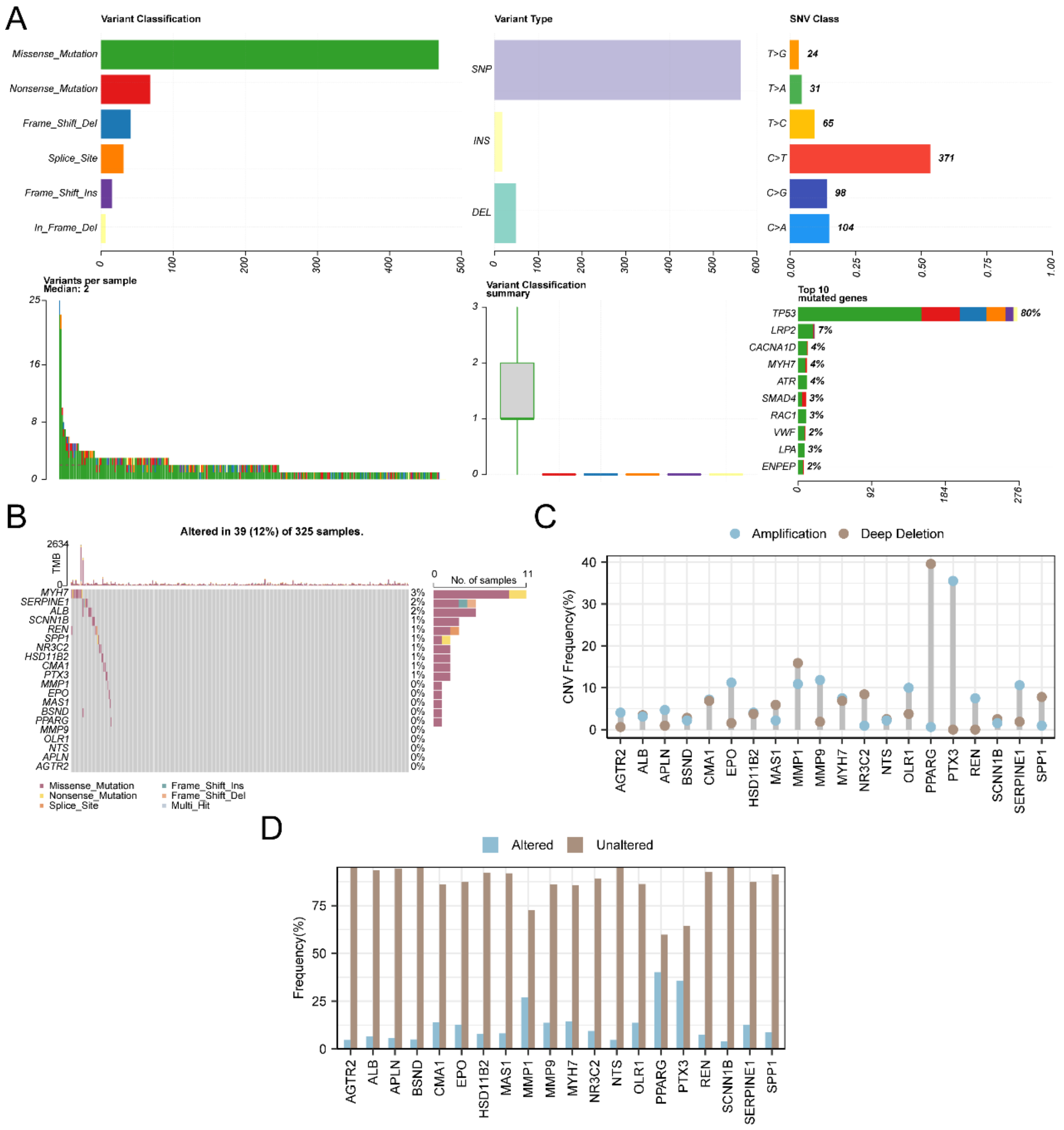

Fig. S2 Mutation analysis of RASRGs in OSCC samples from the TCGA-OSCC dataset. **A** SM analysis of RASRGs. **B** SM analysis of positive and negative top10 | log FC | RASRDEGs. **C-D** CNV analysis of positive and negative top10 | log FC | RASRDEGs.

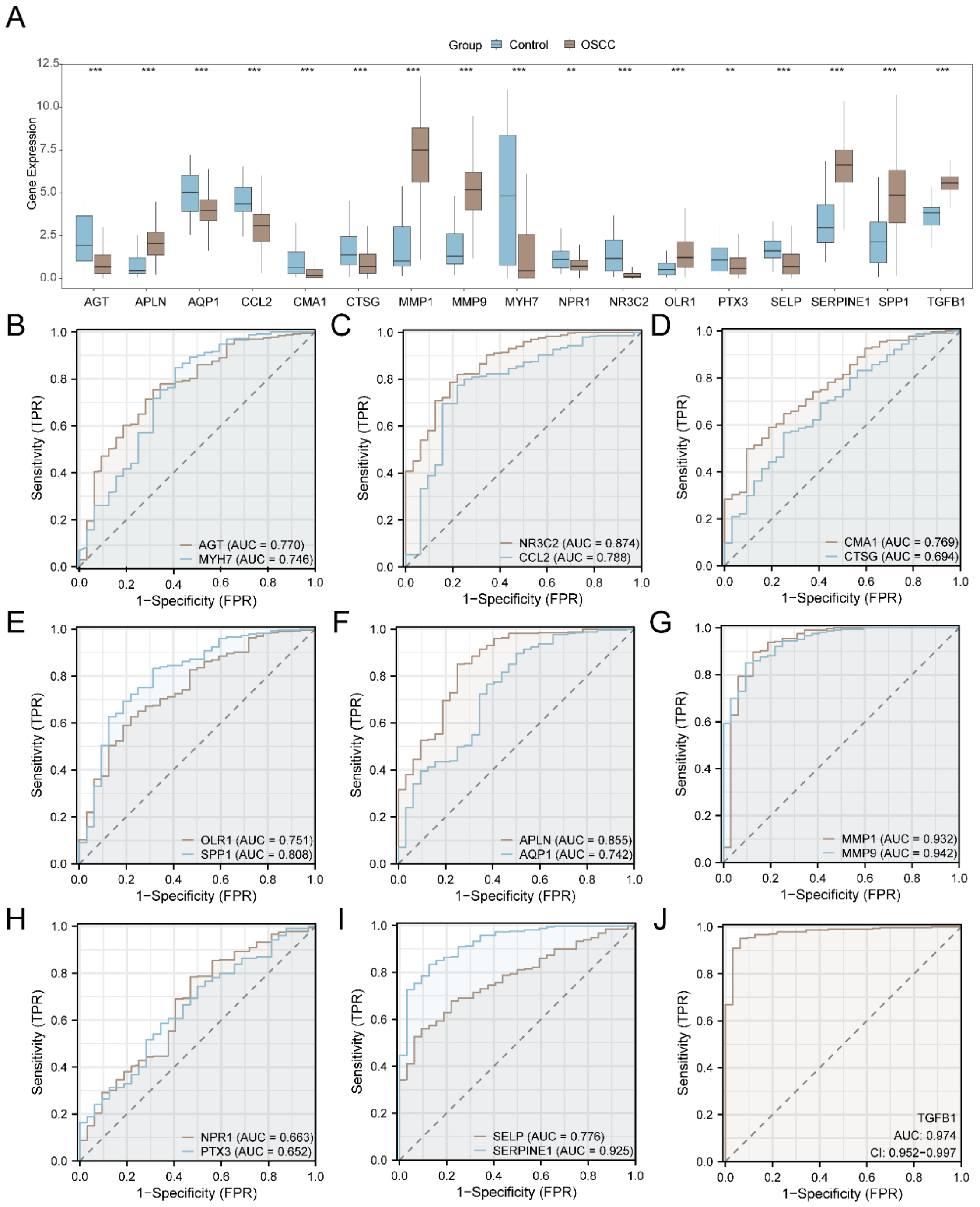

Fig. S3 Differential expression validation and ROC curve analysis of module genes in the TCGA-OSCC dataset. **A** Group comparison diagram of module genes in OSCC and control samples. **B-J** ROC curve of module genes. ** $p < 0.01$, *** $p < 0.001$.

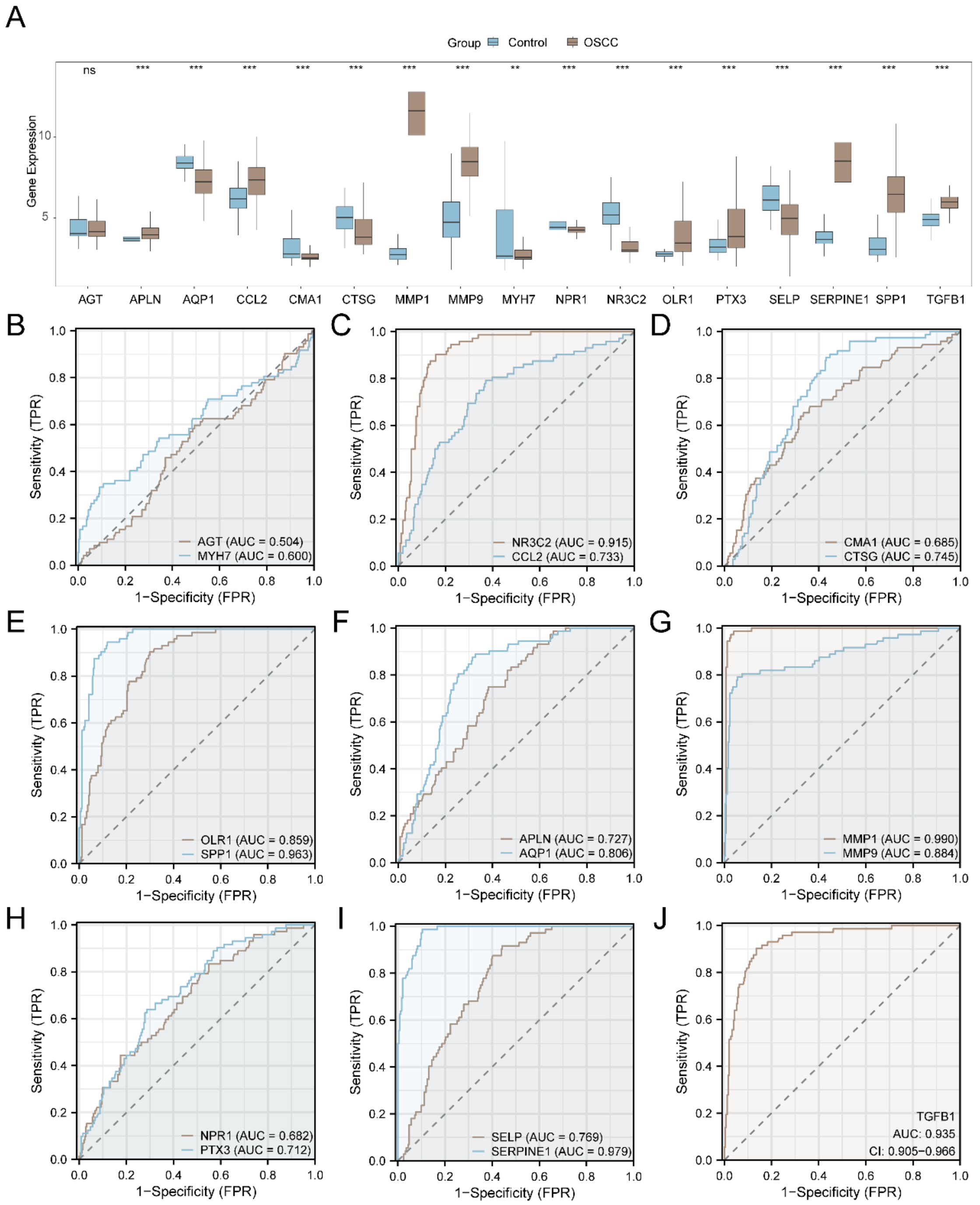

Fig. S4 Differential expression validation and ROC curve analysis of module genes in the combined GEO datasets. **A** Group comparison diagram of module genes in OSCC and control samples. **B-J** ROC curves of module genes. ** $p < 0.01$, *** $p < 0.001$.

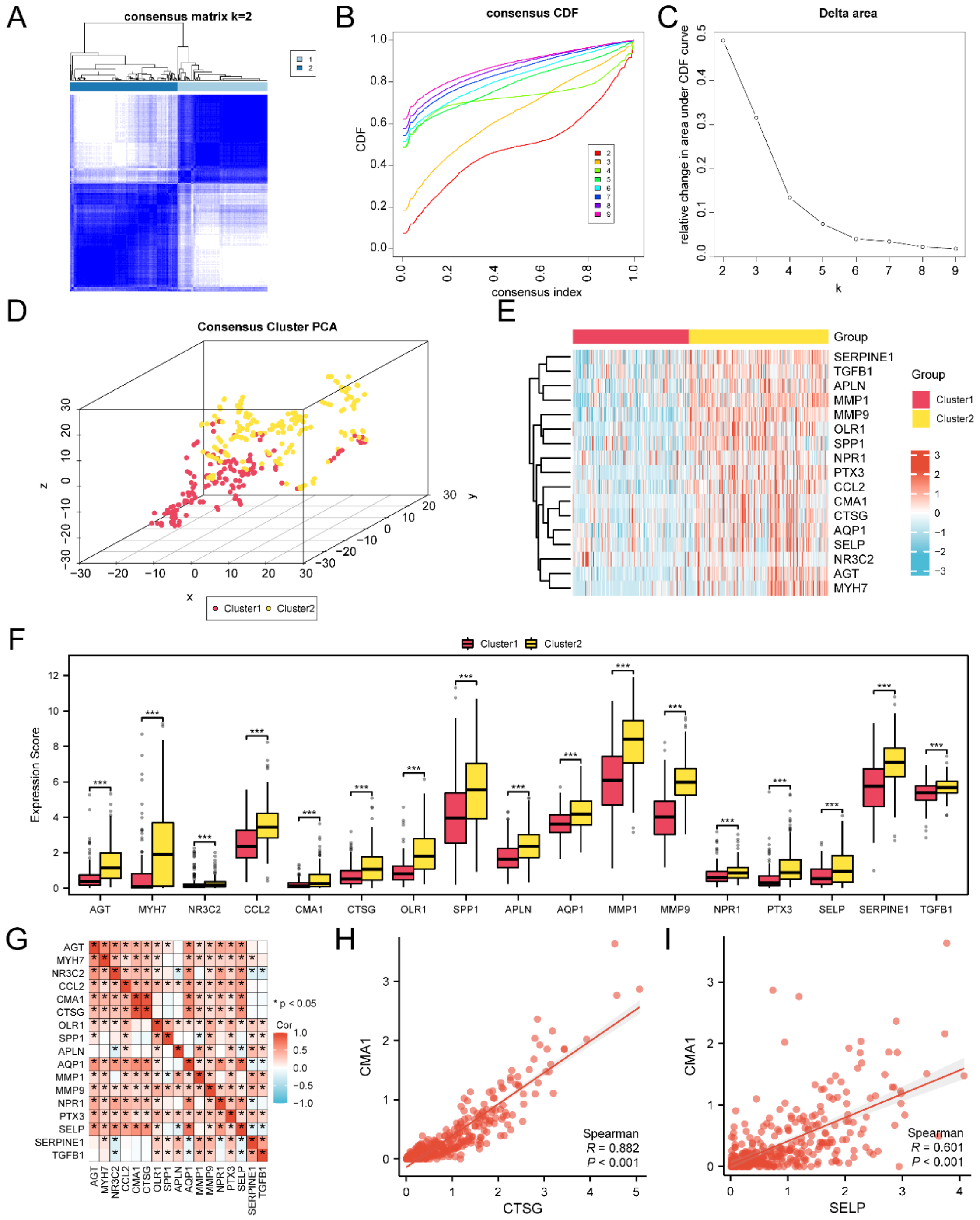

Fig.S5 Consensus clustering analysis of OSCC. **A** Consensus clustering results of OSCC samples from the TCGA-OSCC dataset. **B-C** Consensus cumulative distribution function (CDF) plot (**B**) and Delta plot (**C**) from the consensus clustering analysis. **D** 3D PCA map showing the two disease subtypes of OSCC. **E** Heatmap of module gene expression values across OSCC subtypes. **F** Comparison plot of module gene expression between the two OSCC subtypes. **G** Correlation heatmap of module genes in OSCC samples. **H-I** Scatter plots showing the correlation between module genes, with the top two most positively correlated genes highlighted. ***$p < 0.001$.

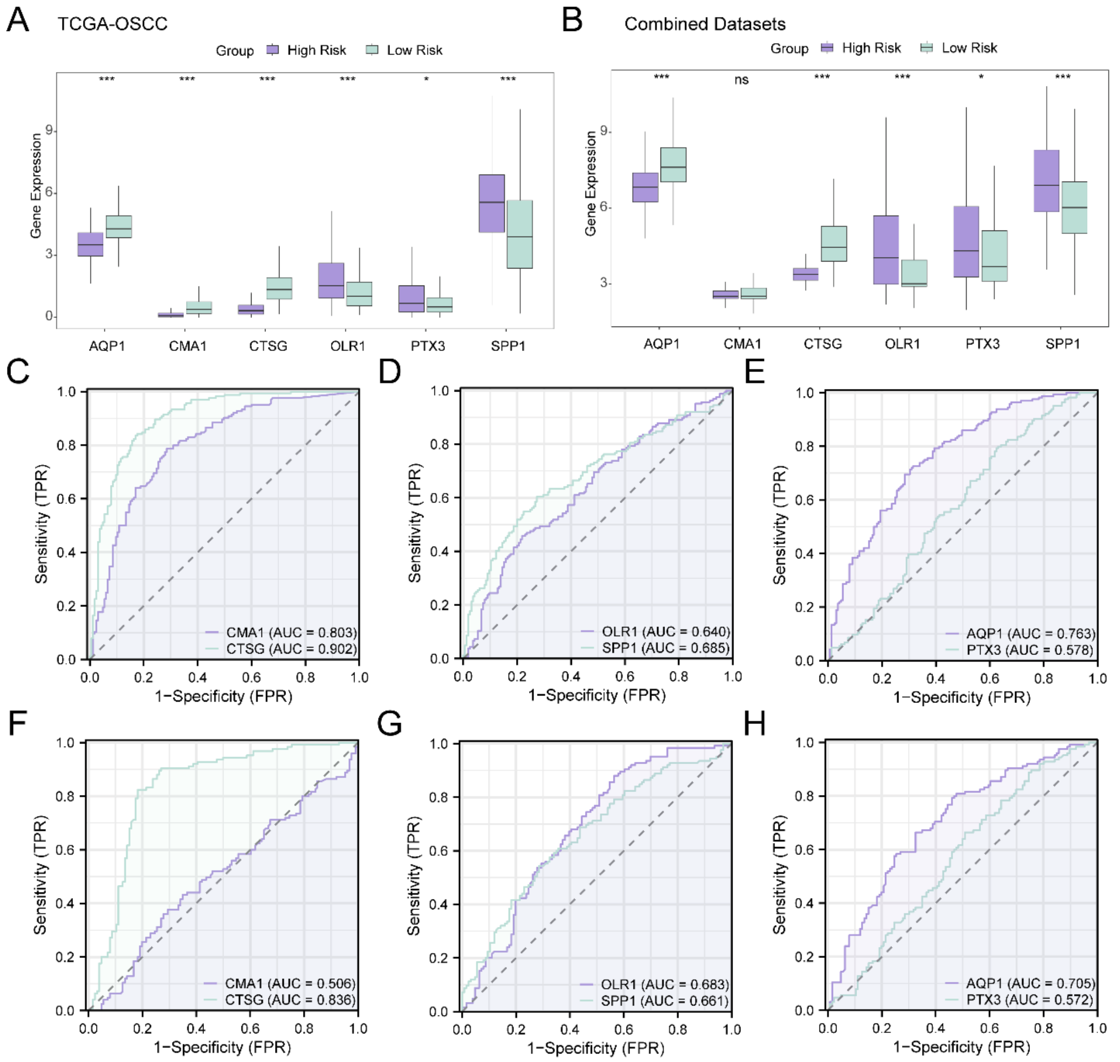

Fig. S6 Differential expression validation and ROC curve analysis of key genes. **A-B** Group comparison plots of six key genes between different OSCC risk groups in the TCGA-OSCC dataset (**A**) and combined GEO datasets (**B**). **C-E** ROC curves for the six key genes in OSCC samples from the TCGA-OSCC dataset. **F-H** ROC curves for the six key genes in OSCC samples from the combined GEO datasets. * $p < 0.05$, ** $p < 0.01$, *** $p < 0.001$.

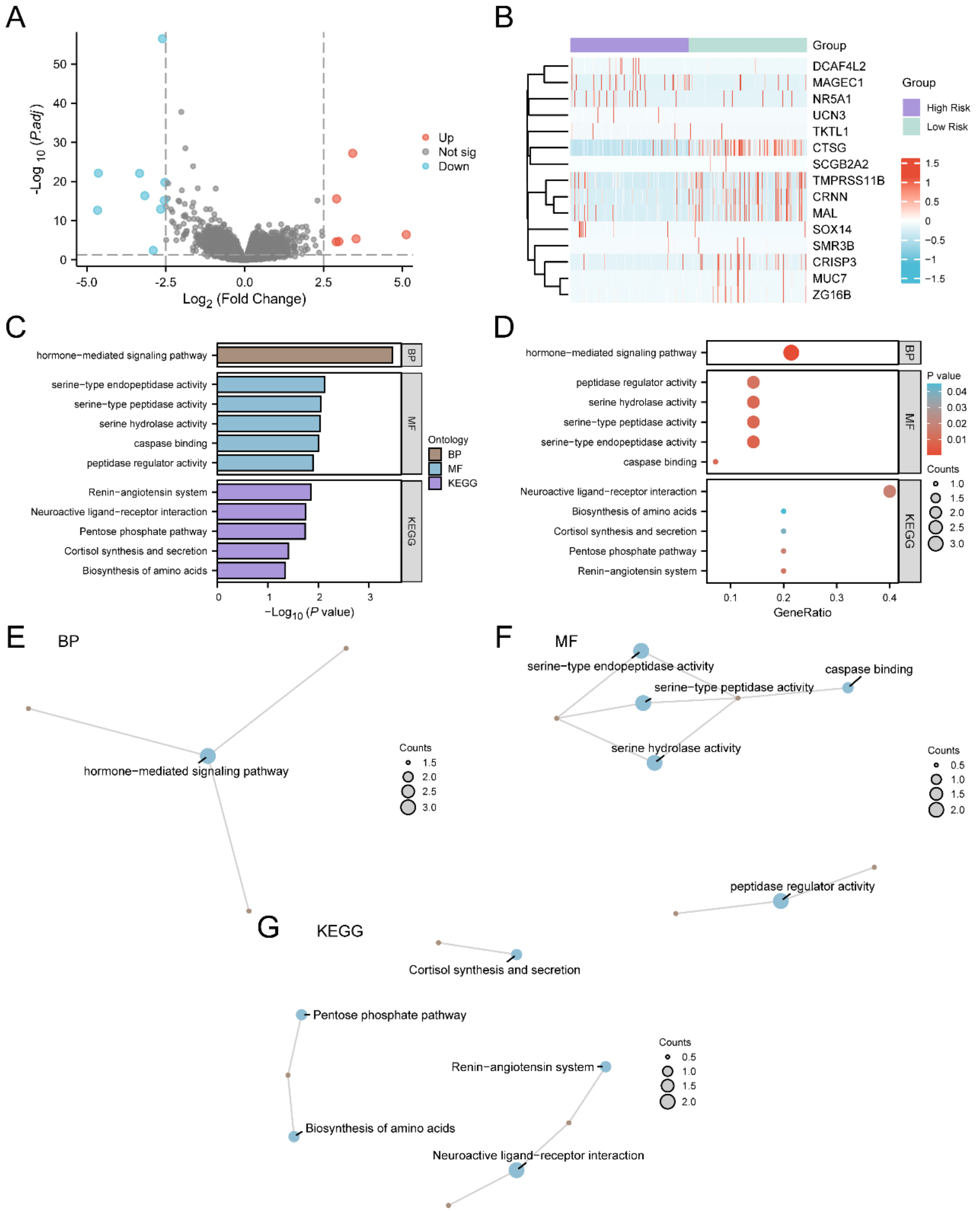

Fig. S7 GO and KEGG enrichment analyses of 15 DEGs among OSCC risk groups. **A** Volcano plot of 15 DEGs B. Expression heat map of DEGs different OSCC risk groups. **C-D** Bar graph (**C**) and bubble plot (**D**) of GO and KEGG pathway enrichment analysis. **E-G** Network diagram of GO and KEGG pathway enrichment analysis results.

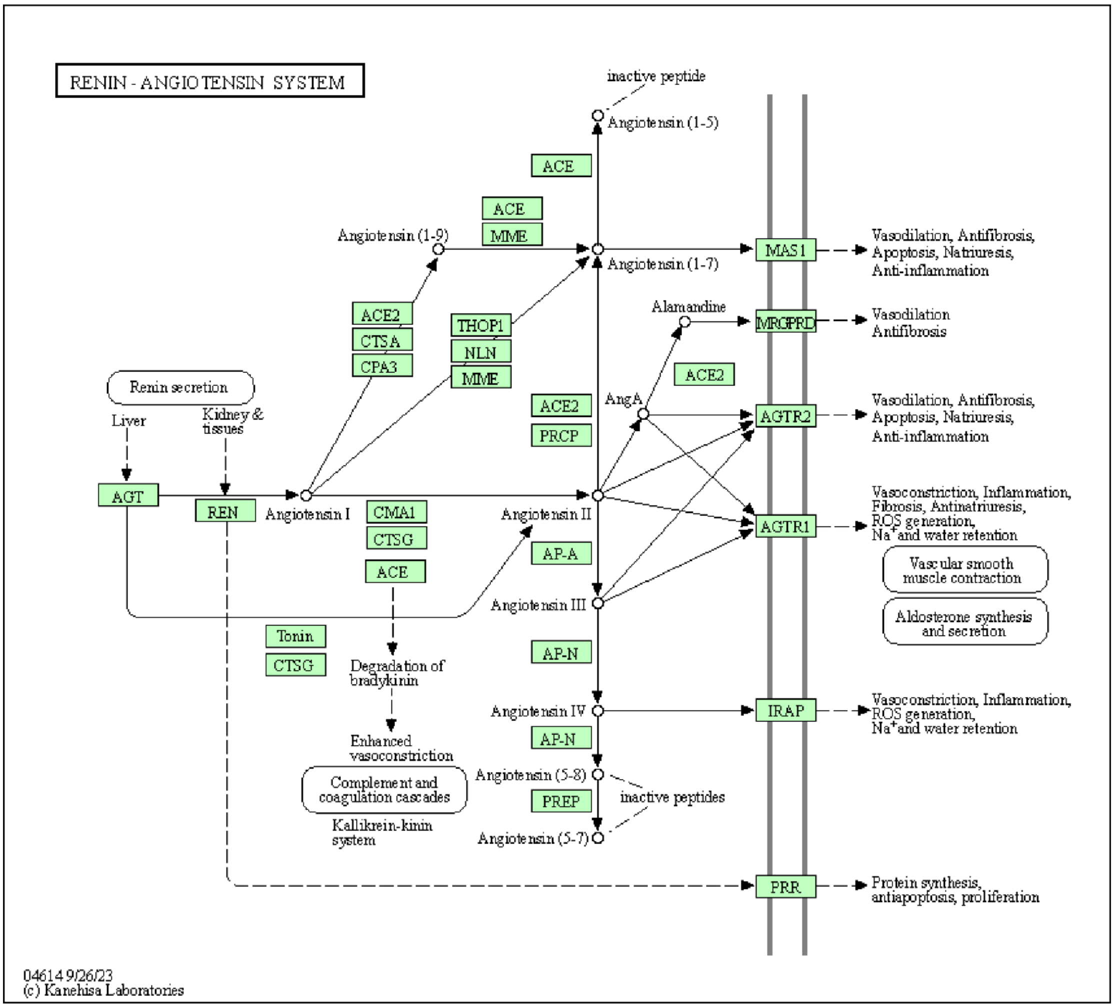

Fig. S8 Visualization of renin-angiotensin system in pathway (KEGG) enrichment analysis.

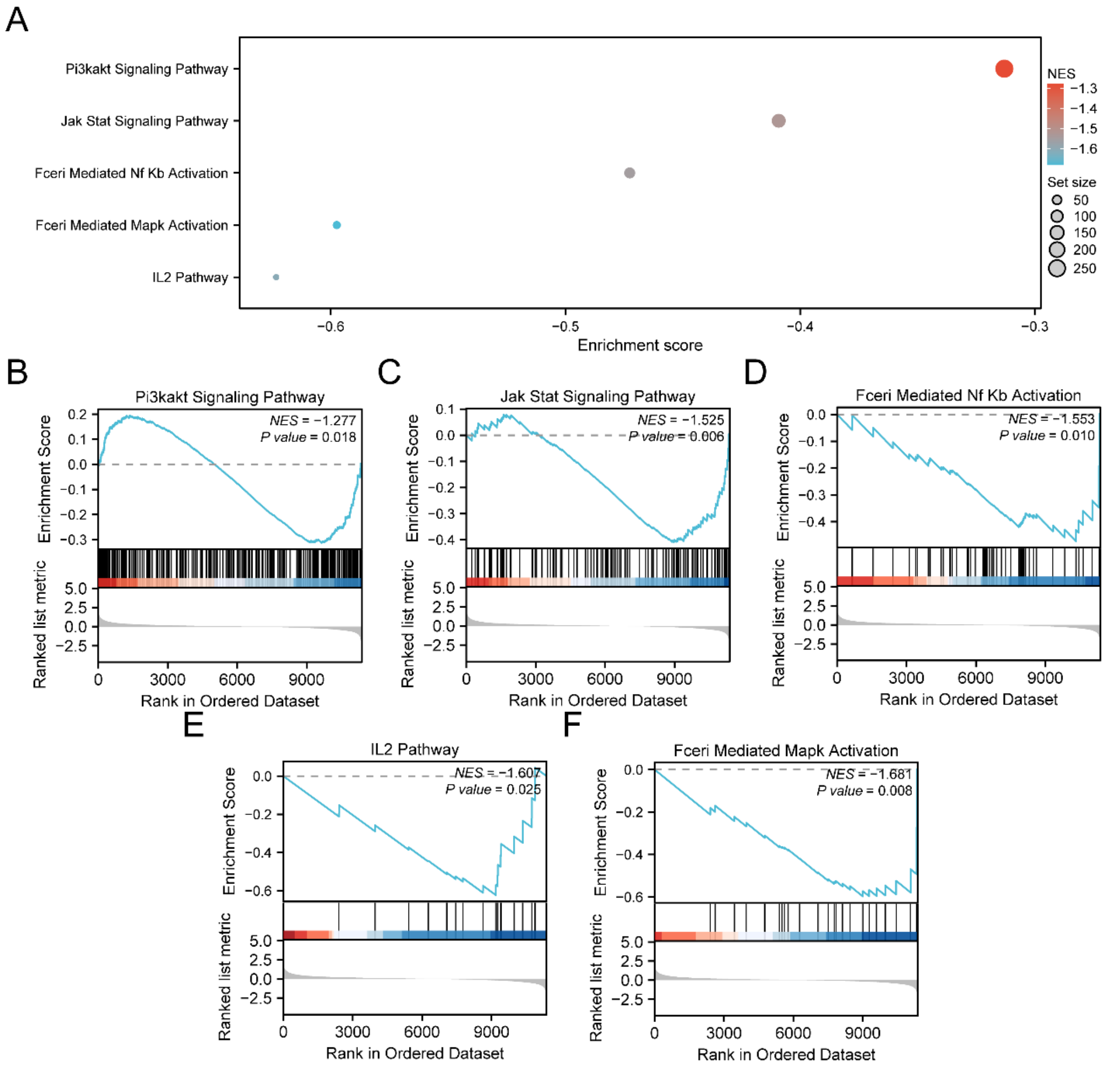

Fig. S9 GSEA of genes in OSCC samples from the TCGA-OSCC dataset. **B-F** Enriched pathway of OSCC in GSEA. All genes in OSCC samples were significantly enriched in PI3K-Akt Signaling Pathway (**B**), JAK-STAT Signaling Pathway (**C**), FceRI mediated NF-κB Activation (**D**), IL-2 Pathway (**E**) and FceRI mediated MAPK Activation (**F**).

Supplemental Tables

Table S1 GEO microarray chip information

	GSE30784	GSE23558	GSE25099
Platform	GPL570	GPL6480	GPL5175
Species	Homo sapiens	Homo sapiens	Homo sapiens
Tissue	Oral Tissue	Oral Tissue	Oral Tissue
Samples in OSCC group	167	27	57
Samples in Control group	45	5	22
Reference	PMID: 18669583	PMID: 22072328<br>PMID: 28433800<br>PMID: 37245006	PMID: 21853135

GEO: Gene Expression Omnibus; OSCC: oral squamous cell carcinoma

Table S2 RASRGs collected from database and literature

RASRGs	AGT, REN, ACE, AGTR1, AGTR2, ATP6AP2, CMA1, LNPEP, ENPEP, ANPEP, ACE2, MME, PRCP, PREP, MAS1, NLN, THOP, CYP11B2, NOS3, NPPA, KNG1, NR3C2, SERPINE1, BDKRB2, RENBP, ADD1, TGFB1, VEGFA, CLCNKB, SLC12A1, INS, ALB, BSND, CLCNKA, TNF, PPARG, KCNJ1, EDN1, GNB3, CASR, MYBPC3, SLC12A3, SCNN1B, SCNN1G, MTHFR, CCL2, APOE, KLKB1, CYP11B1, ADIPOQ, SCNN1A, NPPB, INSR, CRP, F5, AQP2, ERAP1, WNK1, MYH7, IL6, NPHS2, SLC3A1, CTSG, VDR, NPR1, MMP9, CACNA1D, NPHS1, PLAT, IL10, SLC26A3, MAGED2, MAPK1, ADM, ITGB3, CNNM2, CPQ, SLC6A4, VWF, HSD11B2, AGER, XK, EPO, BMP6, KL, LEP, ANG, CCN2, FGF23, TLR4, SPP1, CLTRN, SMAD4, APLN, CD36, CGA, ADRB1, MMP1, CDKN1B, CDKN2B, PTX3, JUN, CYP21A2, PRL, ATR, OLR1, SF1, TNFSF12, MAPK3, MRGPRD, GNAS, TNNI3, TNNT2, PTH, RAN, CD79A, TP53, STAT5A, ADORA2A, AQP1, NPY, RNPEP, CYP17A1, BAX, TIMP1, LPA, NTS, CALCA, GUCY1A1, TBPL1, CXCR4, VCAM1, MC2R, SELP, NOX1, HMOX1, STAT5B, HMGCR, NRF1, PPBP, BDKRB1, CETP, AGTRAP, LDLR, RAC1, ADRB2, SERPINA1, F12, SIRT1, CCR5, UTS2, TMPRSS2, JAK2, LRP2, CTSA

RASRGs: renin-angiotensin system-related genes

Table S3 Clinical characteristics of IHC patient cohort

Patient ID	Gender	Age	Tumor differentiation	Tumor site	TNM Stage	Risk Factors
1	M	39	Moderately differentiated	Tongue	T3N0M0	Leukoplakia (14 years)
2	M	38	Well-differentiated	Left tongue	T1N0M0	Submucosal fibrosis of bilateral buccal mucosa
3	M	32	Well-differentiated	Right tongue	T2N1M0	None
4	M	55	Well-differentiated	Right hard palate	T3N0M0	None
5	M	53	Well-differentiated	Tongue	T2N0M0	Smoking (>30 years)
6	F	52	Moderately-to-well differentiated	Tongue	T4N1M0	None
7	M	74	basaloid subtype	Tongue	T2N0M0	Smoking (>10 years); Alcohol (>20 years)
8	M	66	Well-differentiated	Tongue	T2N0M0	Smoking (>50 years)
9	M	49	Well-differentiated	Tongue	T3N0M0	Smoking (>40 years); Occasional betel nut chewing
10	M	72	Well-differentiated	Tongue	T4N1M0	Smoking (>40 years)
11	F	54	Well-differentiated	Tongue	T2N0M0	None
12	M	27	Well-differentiated	Tongue	T2N0M0	Smoking (>10 years); Betel nut chewing (>1 year)
13	M	50	Well-differentiated	Tongue	T4bN0M0	Smoking and alcohol (20-30 years); Betel nut chewing (1-2 years)
14	M	60	Moderately-to-well differentiated	Tongue	T4N0M0	Smoking and alcohol (>10 years)
15	M	64	Well-differentiated	Tongue	T4N0M0	Smoking (40 years)
16	M	48	Moderately differentiated	Tongue	T2N2bM0	Smoking (>10 years)

Patient ID	Gender	Age	Tumor differentiation	Tumor site	TNM Stage	Risk Factors
17	M	37	Moderately differentiated	Left tongue	T2N0M0	Betel nut chewing (3 years); Smoking (10 years)
18	M	71	Moderately differentiated	Left tongue	T2N0M0	Smoking (30 years)
19	M	83	Well-differentiated	Right buccal mucosa	T4N1M0	Smoking (30 years)
20	F	34	Well-differentiated	Right ventral tongue and right floor of mouth	T4N1M0	Underwent right tongue leucotomy biopsy in 2018

M: male; F: female; T: tumor; N: nodes; M: metastasis

Table S4 RASRDEGs identified in OSCC

RASRDEGs	TGFB1, MMP1, MMP9, HSD11B2, SERPINE1, NR3C2, BSND, AQP1, PPARG, MYH7, AGTRAP, SPP1, SCNN1B, EPO, CCL2, APLN, AGT, CMA1, SCNN1A, PTX3, ANG, CACNA1D, CTSG, REN, NPR1, ADRB1, OLR1, ALB, IL6, SELP, KL, UTS2, AGTR1, SCNN1G, KLKB1, MAS1, CALCA, AGTR2, PPBP, AQP2, SLC3A1, NTS, NPY

RASRDEGs: renin-angiotensin system-related differentially expressed genes; OSCC: oral squamous cell carcinoma

Table S5 Cox analysis for prognostic indicators

Characteristics	Total(N)	Univariate analysis		Multivariate analysis	
		Hazard ratio (95% CI)	P value	Hazard ratio (95% CI)	P value
Age	295	1.42 [1.01 − 2]	4.57e−02	1.51 [1.07 − 2.14]	0.020
Gender	295	0.92 [0.644 − 1.31]	6.48e−01		
N Stage	295	0.787 [0.42 − 1.47]	6.49e−05		
N0	115			Reference	
N1	49			0.76 [0.37 − 1.55]	0.447
N2&3	107			2.13 [1.24 − 3.67]	0.006
NX	24			1.98 [1.08 − 3.61]	0.027
T Stage	295	2.13 [0.752 − 6.03]	2.13e−04		
T1&2	121			Reference	
T3	65			1.74 [0.91 − 3.35]	0.095
T4	109			1.98 [1.01 − 3.86]	0.046
Stage	295	1.85 [0.549 − 6.26]	1.40e−03		
I&II	70			Reference	
III	60			1.36 [0.55 − 3.37]	0.503
IV	165			0.97 [0.38 − 2.47]	0.952
Risk Score	295	2.59 [1.76 − 3.79]	1.11e−06	2.17 [1.44 − 3.28]	< 0.001

T: tumor; N: nodes

Table S6 miRNAs associated with hub genes

mRNA (hub genes)	miRNA
CRNN	hsa-miR-335-5p, hsa-miR-129-2-3p, hsa-miR-146a-5p, hsa-miR-330-3p, hsa-miR-373-3p
TMPRSS11B	hsa-miR-27a-3p
MUC7	hsa-miR-26b-5p, hsa-miR-27a-3p, hsa-miR-517a-3p
SMR3B	hsa-let-7b-5p, hsa-miR-16-5p, hsa-miR-26a-5p, hsa-miR-27a-3p
ZG16B	hsa-miR-335-5p, hsa-let-7b-5p, hsa-miR-124-3p, hsa-miR-129-2-3p, hsa-miR-146a-5p, hsa-miR-212-3p

Table S7 GO and KEGG enrichment analysis of 15 DEGs among OSCC risk groups

ONTOLOGY	ID	Description	Gene Ratio	B/g Ratio	P value
BP	GO:0009755	hormone-mediated signaling pathway	3/14	190/18800	3.41E-04
MF	GO:0004252	serine-type endopeptidase activity	2/14	174/18410	7.50E-03
MF	GO:0008236	serine-type peptidase activity	2/14	191/18410	8.98E-03
MF	GO:0017171	serine hydrolase activity	2/14	195/18410	9.34E-03
MF	GO:0089720	caspase binding	1/14	13/18410	9.84E-03
MF	GO:0061134	peptidase regulator activity	2/14	230/18410	1.28E-02
KEGG	hsa04614	Renin-angiotensin system	1/5	23/8164	1.40E-02
KEGG	hsa04080	Neuroactive ligand-receptor interaction	2/5	362/8164	1.79E-02
KEGG	hsa00030	Pentose phosphate pathway	1/5	30/8164	1.82E-02
KEGG	hsa04927	Cortisol synthesis and secretion	1/5	65/8164	3.92E-02
KEGG	hsa01230	Biosynthesis of amino acids	1/5	75/8164	4.51E-02

GO: Gene Ontology; BP: biological process; MF: molecular function; KEGG: Kyoto Encyclopedia of Genes and Genomes; DEGs: differentially expressed genes; OSCC: oral squamous cell carcinoma

Table S8 GSEA of genes in OSCC samples from the TCGA-OSCC dataset

ID	Set Size	Enrichment Score	NES	p value
REACTOME_REGULATION_OF_GENE_EXPRESSION_IN_BETA_CELLS	17	8.15E-01	2.11E+00	2.01E-03
WP_CILIOPATHIES	119	5.57E-01	2.05E+00	1.98E-03
WP_GENES_RELATED_TO_PRIMARY_CILIUM_DEVELOPMENT_BASED_ON_CRISPR	66	6.00E-01	2.03E+00	1.96E-03
KEGG_MATURITY_ONSET_DIABETES_OF_THE_YOUNG	21	7.19E-01	1.96E+00	2.07E-03
WP_RETINOBLASTOMA_GENE_IN_CANCER	68	5.62E-01	1.91E+00	1.95E-03
REACTOME_OLFACTORY_SIGNALING_PATHWAY	51	5.83E-01	1.89E+00	3.82E-03
REACTOME_REGULATION_OF_GENE_EXPRESSION_BY_HYPOXIA_INDUCIBLE_FACTOR	10	8.37E-01	1.88E+00	1.94E-03
REACTOME_REGULATION_OF_BETA_CELL_DEVELOPMENT	34	6.25E-01	1.87E+00	5.95E-03
PID_PLK1_PATHWAY	30	6.35E-01	1.86E+00	8.02E-03
REACTOME_CELL_CYCLE_CHECKPOINTS	168	4.83E-01	1.86E+00	1.98E-03
REACTOME_REPRODUCTION	59	5.61E-01	1.86E+00	1.96E-03
REACTOME_MITOTIC_SPINDLE_CHECKPOINT	73	5.39E-01	1.85E+00	1.94E-03
REACTOME_HOMOLOGY_DIRECTED_REPAIR	62	5.53E-01	1.85E+00	1.97E-03
WP_GASTRIC_CANCER_NETWORK_2	22	6.67E-01	1.83E+00	6.20E-03
WP_BILE_ACIDS_SYNTHESIS_AND_ENTEROHEPATIC_CIRCULATION	10	8.14E-01	1.83E+00	3.88E-03
WP_PI3KAKT_SIGNALING_PATHWAY	252	-3.13E-01	-1.28E+00	1.78E-02
KEGG_JAK_STAT_SIGNALING_PATHWAY	116	-4.09E-01	-1.52E+00	5.99E-03
REACTOME_FCERI_MEDIATED_NF_KB_ACTIVATION	55	-4.73E-01	-1.55E+00	1.02E-02
BIOCARTA_IL2_PATHWAY	18	-6.23E-01	-1.61E+00	2.50E-02
REACTOME_FCERI_MEDIATED_MAPK_ACTIVATION	24	-5.97E-01	-1.68E+00	7.62E-03
